# Entwicklung des Concept-Inventory CCCI-422 zu den naturwissenschaftlichen Grundlagen des Klimawandels

**DOI:** 10.1007/s40573-023-00159-8

**Published:** 2023-05-04

**Authors:** Thomas Schubatzky, Rainer Wackermann, Carina Wöhlke, Claudia Haagen-Schützenhöfer, Marko Jedamski, Hannes Lindemann, Kai Cardinal

**Affiliations:** 1 Institut für Fachdidaktik und Institut für Experimentalphysik, Universität Innsbruck, Innsbruck, Österreich; 2 Fakultät für Physik und Astronomie, AG Didaktik der Physik, Ruhr-Universität Bochum, Bochum, Deutschland; 3 Institut für Physik, Physikdidaktik, Karl-Franzens Universität Graz, Graz, Österreich; 4 Fakultät für Physik, Didaktik der Physik, Universität Duisburg-Essen, Duisburg-Essen, Deutschland

**Keywords:** Deutsch: Concept Inventory, Klimawandel, Schülervorstellungen, Testentwicklung, Validierung, Konzeptuelles Verständnis, English: concept inventory, Climate change, Students’ conceptions, Test development, Validation, Conceptual understanding

## Abstract

Der aktuelle Klimawandel stellt unsere Gesellschaft, besonders zukünftige Generationen, vor große Herausforderungen. Ein grundlegendes Verständnis des Klimawandels kann nicht nur Jugendlichen dabei helfen, falsche oder widersprüchliche Darstellungen besser einzuschätzen, um so am gesellschaftlichen Diskurs zum Thema Klimawandel teilhaben zu können. Um Aussagen über das Verständnis von zentralen fachlichen Inhalten zum Klimawandel zuverlässig treffen zu können, braucht es geeignete Testinstrumente. Für die Entwicklung eines Concept Inventories zu den naturwissenschaftlichen Grundlagen des Klimawandels (den wir CCCI-422 nennen) wurden zentrale fachliche Inhalte identifiziert, offene Aufgaben entwickelt und schließlich Distraktoren und Attraktoren aus insgesamt über 50 Interviews abgeleitet. Anschließend wurde der Concept Inventory in mehreren Pilotierungs- und Validierungsstudien eingesetzt, um so unterschiedliche Argumente, die für den Einsatz des CCCI-422 sprechen, zu sammeln. Es wurde etwa eine Teilstudie durchgeführt, um ein Argument für die externe Validität der Testwertinterpretation zu formulieren. Dabei zeigten sich theoretisch angenommene Unterschiede auch in den Personenfähigkeiten. In zwei weiteren Teilstudien konnten zusätzlich Argumente für die Instruktionssensitivität des Concept Inventories formuliert werden. Als Argument für die Konstruktvalidität lässt sich zudem auf Basis von Dimensionsanalysen eine eindimensionale sowie fünfdimensionale Struktur ableiten. Diese fünf Dimensionen entsprechen den fünf theoretisch abgeleiteten Inhaltsbereichen, wobei tendenziell *Unterschied Wetter – Klima* der leichteste und *Atmosphäre unserer Erde* der schwerste Inhaltsbereich sind. Wir plädieren daher dafür, dass der CCCI-422 abhängig vom Einsatzzweck als eindimensionaler oder fünfdimensionaler Concept Inventory genutzt werden kann. In diesem Beitrag wird der entwickelte Concept Inventory inklusive seinem Entstehungsprozess vorgestellt, der als Onlinematerial zur Nutzung zur Verfügung gestellt wird.

**Zusatzmaterial online:**

Zusätzliche Informationen sind in der Online-Version dieses Artikels (10.1007/s40573-023-00159-8) enthalten.

## Abstract (en)

Current climate change poses major challenges to our society. A basic understanding of climate change can particularly help people to better assess false or contradictory representations to be able to participate in the social discourse on the topic of climate change. To reliably make statements about the understanding of core content areas on climate change, suitable test instruments are needed.

For the development of a concept inventory regarding the scientific principles of climate change (which we call CCCI-422), core content areas were identified, open-ended tasks were developed, and finally distractors and attractors were derived from a total of over 50 interviews. Subsequently, the Concept Inventory was used in several piloting and validation studies, thus collecting different arguments in favor of using the CCCI-422. For example, one study was conducted to formulate an argument for the external validity of the test score interpretation. Theoretically hypothesized differences were also shown in differences in person abilities. In two further studies, arguments for the instructional sensitivity of the Concept Inventory could be formulated. Furthermore, as an argument for construct validity, a one-dimensional as well as a five-dimensional structure can be derived based on dimensional analyses. The five dimensions correspond to the five theoretically derived content areas, with *difference weather—climate* tending to being the easiest and *atmosphere of our Earth* being the most difficult content area. Therefore, we argue that the CCCI-422 can be used as a one-dimensional or five-dimensional Concept Inventory depending on the purpose of use. This paper presents the developed Concept Inventory including its development process, which is provided as online material for use.

## Einleitung

SchülerInnen wachsen aktuell in unsicheren, krisenbehafteten Zeiten auf. Die sozialen, ökologischen, wirtschaftlichen, kulturellen und umweltbezogenen Auswirkungen des globalen Klimawandels sind eine Herausforderung, die ihren Alltag und ihr Umfeld immer stärker beeinflusst (Rousell und Cutter-Mackenzie-Knowles 2020; Selby und Kagawa 2010). Allerdings gibt es immer noch eine oberflächliche Kontroverse um den Klimawandel, die zu Verwirrung über die naturwissenschaftlichen Grundlagen des Klimawandels führen kann, da der Klimawandel in den Medien manchmal falsch dargestellt wird (Coan et al., 2021; McCright und Dunlap 2011). Einige Studien berichten, dass SchülerInnen mehr über den Klimawandel durch die Medien erfahren als durch formalen Unterricht, was zur Entwicklung von nicht angemessenen Vorstellungen über den Klimawandel beitragen kann (z. B. Liu et al. 2015; Miller 2012; Nation 2017; Nation und Feldman 2021). Solche weit verbreiteten alternativen Vorstellungen werden als große Hindernisse für angemessene Einstellungen und Handlungsbereitschaften angesehen, die im naturwissenschaftlichen Unterricht im Allgemeinen (Vosniadou 2009), aber eben auch im Unterricht über den Klimawandel (Niebert 2010) überwunden werden müssen. SchülerInnen sollten eine Klima-Grundbildung erlangen, was neben entsprechenden Einstellungen und Fähigkeiten auch bedeutet, dass sie die grundlegenden Prinzipien des Klimasystems und des Treibhauseffekts auf der Erde verstehen (USGCRP 2009). Dementsprechend stellt ein Verständnis der naturwissenschaftlichen Grundlagen des Klimawandels eine der Säulen der *Climate Literacy* dar (USGCRP 2009), die es beispielsweise ermöglicht, informierte Einstellungen ausbilden zu können. Um unterrichtliche Angebote zu evaluieren und SchülerInnen zu unterstützen, verbreitete alternative Lernendenvorstellungen im naturwissenschaftlichen Unterricht zu überwinden, ist eine gründliche Kenntnis des gegenwärtigen Verständnisses der SchülerInnen (und damit deren Vorstellungen) unerlässlich. Eine grundsätzliche Fragestellung fachdidaktischer Forschung ist, inwiefern derartige Vorstellungen auch von einer größeren Anzahl an SchülerInnen zuverlässig erhoben werden können, etwa um Lernfortschritte und -zuwächse messen zu können. Concept Inventories (CI) stellen hier einen etablierten Zugang dar, und obwohl es in der Vergangenheit bereits einige Anläufe gab, ein CI zu den naturwissenschaftlichen Grundlagen des Klimawandels zu entwickeln (z. B. Arslan et al. 2012; Jarrett und Takacs 2020; Keller 2006; Lambert et al. 2012; Lombardi et al. 2013), liegt bis dato noch kein CI in deutscher Sprache vor. Das Ziel dieser Studie war es daher, einen aktuellen Klimawandel-CI (in einem ersten Schritt in deutscher Sprache) für den Einsatz ab Ende der Sekundarstufe I zu entwickeln, der aus früheren Studien und bestehender Literatur zum Verständnis des Klimawandels bei SchülerInnen abgeleitet ist. In diesem Artikel wird die Entwicklung dieses CIs dargestellt.

Das entwickelte Testinstrument soll konzeptuelles Verständnis der naturwissenschaftlichen Grundlagen des Klimawandels erheben, wobei es anhand der Alternativantworten zusätzlich möglich sein soll, auf alternative Lernendenvorstellungen schließen zu können. Dadurch soll es in zukünftigen Studien möglich sein, typische Lernverläufe über die naturwissenschaftlichen Grundlagen des Klimawandels beschreiben zu können. Wir stellen daher auch mögliche Einsatzzwecke und Testwertinterpretationen für diesen CI dar, die wir durch spezifische Validitätsargumente stützen. In den nächsten Kapiteln gehen wir daher zunächst auf prinzipielle Vorteile und Limitationen von CIs ein. Wir beschreiben die testtheoretische Rahmung der Entwicklung dieses CIs, die sich im Wesentlichen an den Richtlinien der AERA (2014) bzw. dem Artikel von Meinhardt et al. (2018) orientiert, und legen anschließend das Validitätskonzept, welches wir in diesem Artikel verfolgen, offen. Danach beschreiben wir die unterschiedlichen Entwicklungsschritte des CCCI-422 und leiten daraus ab, welche Argumente wir für die Interpretation der Antwortverhalten aus unserer Sicht finden konnten, inklusive einer Erhebung mit knapp 800 Lernenden aus Deutschland und Österreich.

## Concept Inventories in naturwissenschaftsdidaktischer Forschung

Ein CI ist ein Multiple-Choice-Instrument, das entwickelt wird, um das konzeptionelle Verständnis von SchülerInnen zu bestimmten fachlichen Inhaltsbereichen zu erheben (Lindell et al. 2007). Das übergeordnete Ziel ist also, Lernstände zuverlässig diagnostizieren und Lernzuwächse messen zu können. Dazu werden Aufgaben zu zentralen fachlichen Inhaltsbereichen erstellt (Jorion et al. 2015), wobei jedes Item aus einem Attraktor (richtige Antwort) sowie mehreren Distraktoren (falsche Antworten) besteht. Die Distraktoren basieren dabei auf häufig auftretenden alternativen Vorstellungen von Lernenden (Sadler et al. 2009). Die Erhebung von Lernendenvorstellungen wird insbesondere dazu als essenziell betrachtet, konzeptionelles Verständnis zu diagnostizieren (Sadler et al. 2009). Das Identifizieren von Lernendenvorstellungen ist daher von zentraler Bedeutung für die Beschreibung und Messung des Verständnisstands eines Schülers oder einer Schülerin und ist normalerweise eines der beabsichtigten Ergebnisse eines CIs (Sands et al. 2018). Ein Knackpunkt in der Entwicklung eines CIs liegt also in der Auswahl geeigneter Aufgaben mit geeigneten Distraktoren (Pérez García et al. 2016).

Der große Vorteil von CIs ist, dass sie relativ zuverlässig Lernzuwächse messen können, welche über die Entwicklung von rein deklarativem Wissen hinausgehen, und dass diese Lernzuwächse auch noch inhaltlich beschrieben werden können, etwa anhand der Änderung von Lernendenvorstellungen (Sands et al. 2018). Es lassen sich aber auch Gefahren und Schwierigkeiten im Umgang mit CIs formulieren: Die meisten CIs nutzen Multiple-Choice oder Single-Choice Strukturen, sodass die Gefahr besteht, dass Lernende durchaus auch Antworten aus anderen Gründen wählen als von den TestentwicklerInnen intendiert. Außerdem stellt sich beim Einsatz von CIs die Frage, ob diese aufgrund der Formulierungen eher das intuitive Verständnis von Lernenden messen anstelle eines tiefergehenden konzeptuellen Verständnisses (Huffman und Heller 1995).

Nichtsdestotrotz werden CIs seit langem eingesetzt. Seit Veröffentlichung des ersten dokumentierten CI, dem Force Concept Inventory (FCI) (Hestenes et al. 1992) wurden bereits zahlreiche weitere CIs für die unterschiedlichsten Inhaltsgebiete entwickelt (z. B. Hestenes et al. 1992; Ivanjek et al. 2021; Keller 2006). Die Entwicklung folgt dabei einem iterativen Vorgehen (Nelson et al. 2007; Porter et al. 2014), die sich auch in den Richtlinien der AERA zur Instrumententwicklung wiederfinden:

1. Erklärung zum Zweck und zur beabsichtigten Verwendung des CIs: Diesem Zweck sind wir teils bereits in der Einleitung nachgekommen, greifen diesen jedoch in der Diskussion noch einmal auf, um diesen Punkt abschließend zu bewerten.
2. Definition der Konzepte und Inhaltsbereiche (auf Basis von Literatur und/oder ExpertInneninterviews), die anhand des CI getestet werden sollen. Diesen Punkt greifen wir im nächsten Kapitel auf.
3. Die Entwicklung von Fragen durch Literaturrecherche als auch Antworten durch die Analyse von Lernprodukten oder Interviews. Diesen Punkt greifen wir im Abschnitt *Entwicklung offener Antworten und Ableitung von Antwortalternativen *auf, sowie in Schubatzky et al. (2022) und Wackermann et al. (2021, 2022).
4. Die iterative Pilotierung des Testinstruments und formulieren von Validitätsargumenten so lange, bis die EntwicklerInnen aus ihrer Sicht genügend Argumente für die Testqualität gesammelt haben. Eine abschließende Bewertung dieses Punktes findet sich in der Diskussion dieses Artikels.

Obwohl die Entwicklung der meisten CIs demselben prinzipiellen Vorgehen wie eben beschrieben folgt, zeigen sich in Entwicklungsprozessen dennoch Unterschiede. In einer vergleichenden Analyse der Entwicklung von zwölf unterschiedlichen CIs zeigt sich etwa, dass insbesondere die Distraktoren der Items auf unterschiedliche Arten entwickelt wurden (Lindell et al. 2007). Während die Antworten mancher Tests auf Schüleraussagen basierten, gründeten sich andere auf die Ideen von ExpertInnen, andere wiederum auf beides. Für die Entwicklung, den Einsatz und die Interpretation von CIs gilt es also, den Entwicklungsprozess des Testinstruments zu kennen und diesen auf Entwicklerseite ausreichend darzustellen. Das Ziel dieses Artikels ist es daher, die Entwicklung des Concept Inventory CCCI-422 darzustellen und Argumente, die für dessen Einsatz sprechen, aber auch Herausforderungen, aufzuzeigen.

Bei der Entwicklung dieses CIs orientierten wir uns aus testtheoretischer Sicht an Richtlinien der AERA (2014), die in dem Werk *Standards for Educational and Psychological Testing* Richtlinien für die Entwicklung von Messinstrumenten beschreiben. Den ersten Schritt in der Entwicklung von Testinstrumenten als notwendige Voraussetzung für eine valide Testwertinterpretation stellt nach den AERA-Richtlinien neben der Angabe von Nutzungsabsichten die Festlegung und Definition des zu messenden Konstrukts dar. Dieser Forderung kommen wir in den nächsten Kapiteln nach, in dem die abzudeckenden Konzepte und Inhaltsbereiche beschrieben werden. Danach legen wir offen, welchem Verständnis von Lernendenvorstellungen wir in diesem Artikel folgen und welche Vorstellungen aus der Literatur bereits bekannt sind. Basierend auf diesen Beschreibungen formulieren wir eine Definition der anhand des CCCI-422 identifizierten Personenfähigkeiten.

## Fachlicher Hintergrund und Definition der abgedeckten Inhaltsbereiche und Konzepte

Der Klimawandel stellt ein interdisziplinäres Thema dar, welches von unterschiedlichen Seiten beleuchtet werden kann. Es kann daher nie eine einzige „Liste“ an zentralen naturwissenschaftlichen Inhalten oder Ideen geben, die für alle Betrachtungsweisen des Klimawandels immer die gleiche Relevanz besitzen. Zusätzlich ist an dieser Stelle hervorzuheben, dass der CCCI-422 durch die Biografien der EntwicklerInnen bedingt aus einer primär physikalischen Perspektive heraus entwickelt worden ist und dies sich auch in den Inhaltsbereichen widerspiegelt, auch wenn versucht worden ist, einen allgemein naturwissenschaftlichen Blick in der Entwicklung einzunehmen. Wenn es um ein basales Grundverständnis des Klimasystems der Erde und des aktuellen Klimawandels aus naturwissenschaftlicher Sicht geht, kristallisieren sich jedoch einige Konzepte und Inhaltsbereiche heraus, die als wesentliche Voraussetzungen für Verständnis gesehen werden können. Basierend auf Fachliteratur (z. B. Dessler 2021; Eyring et al. 2021; Jarrett et al. 2011; Stocker 2011), bereits bestehenden Rahmenkonzepten (z. B. grundlegende Prinzipien des Klimasystem der Erde nach USGCRP 2009) und Interviews mit KlimawissenschaftlerInnen in Österreich und der Schweiz (*N* = 8) sind wir zu dem Schluss gekommen, dass die folgenden fünf Themenbereiche im naturwissenschaftlichen Unterricht zum Thema Klimawandel aufgegriffen werden sollten und daher auch Teil des CCCI-422 sind. Aus der Recherche sowie den ExpertInneninterviews kristallisierte sich zudem heraus, dass auch das Wesen der Klimawissenschaften (fünftes Prinzip aus USGCRP 2009) einen zentralen Inhaltsbereich darstellt, der jedoch in dieser Version des CCCI-422 noch nicht abgedeckt wird, da der CI dadurch einen zusätzlichen, anderen Fokus erhalten würde. Die in den nächsten Abschnitten folgenden inhaltlichen Beschreibungen haben nicht die Absicht, alle Details aus physikalischer Sicht zu beschreiben, sondern sollen das zu messende Konstrukt beschreiben, um eine anschließende Operationalisierung zu ermöglichen. Die folgenden Beschreibungen der Inhaltsbereiche beziehen sich also auf fachwissenschaftliche Werke (Dessler 2021; Eyring et al. 2021; Stocker 2011), bestehende Rahmenkonzepte (USGCRP 2009) sowie Interviews mit KlimawissenschaftlerInnen in Österreich und der Schweiz.

### Die Atmosphäre unserer Erde

Ein grundlegendes Verständnis von Größe, Aufbau und Zusammensetzung der Erdatmosphäre stellt eine wichtige Basis für ein Verständnis des Klimawandels dar. Es geht darum, zu verstehen, dass die Atmosphäre unserer Erde eine dünne Gashülle ist, deren Dichte nach oben hin schnell abnimmt. Es geht auch darum, dass man die Atmosphäre gedanklich in verschiedene Schichten wie Tropo- oder Stratosphäre unterteilen kann. Relevant für das Wetter- und Klimageschehen sind aber nur die Atmosphärenschichten, die der Erdoberfläche sehr nah sind, weil diese bereits den weitaus größten Teil der Luftmenge beinhalten und weil die Erwärmung der Atmosphäre von Richtung des Erdbodens ausgeht. Zentral ist auch, dass die Atmosphäre in ihrer Zusammensetzung homogen erscheint, es muss also genügend Konvektion und Turbulenz geben, um die Gase verschiedener Dichte ständig zu durchmischen.

Außerdem geht es darum, den Anteil an Treibhausgasen in der Atmosphäre einschätzen zu können, die nur in Spuren vorkommen, und ihre entscheidende Eigenschaft zu kennen: Für sichtbares Licht durchlässig zu sein, aber mit Infrarotstrahlung zu wechselwirken.

### Der Unterschied zwischen Wetter und Klima

Hat man erst einmal eine Idee davon, wie die Atmosphäre oder die Luft, die uns umgibt, generell aufgebaut ist, kann man sich Fragen darüber stellen, wie der Zustand der Atmosphäre bzw. Luft an einem bestimmten Ort zu einem bestimmten Zeitpunkt beschrieben werden kann. Dies führt zum Begriff Wetter. Wetter ist das, was wir sehen und spüren, wenn wir nach draußen gehen, etwa Regen, Sonnenschein, kalt oder windig.

Durch Messung bestimmter Parameter wie zum Beispiel der Temperatur lässt sich das Wetter unmittelbar bestimmen, das Klima jedoch nicht. Unter Klima wird etwas Längerfristiges verstanden, das sich durch statistische Werte wie die mittlere Temperatur aber auch deren auftretende Schwankungen (Streuung, Extremwerte), beschreiben lässt. Aber nicht nur auf einer zeitlichen Skala sind diese Unterschiede festzuhalten, sondern auch auf einer räumlichen: So kann von einem globalen Klima gesprochen werden, ein globales Wetter gibt es jedoch nicht. Und während es schwierig ist, das Wetter (etwa die Temperatur) an einem Ort mehr als 10 Tage vorauszusagen, ist es anhand von Klimamodellen sehr wohl möglich, Klimaentwicklungen (etwa mittlere Globaltemperaturen) für Jahrzehnte zuverlässig zu projizieren.

### Das Klima als System

Um das Klima und dessen Veränderungen zu verstehen, reicht es nicht, sich auf Betrachtungen der Atmosphäre zu beschränken. Das Klima unserer Erde kann als System beschrieben werden, in dem unterschiedliche Bestandteile miteinander wechselwirken. Zwischen diesen wird insbesondere Energie, aber auch Materie (etwa Kohlenstoff) ausgetauscht. Dabei verändert sich das Klimasystem über die Zeit unter dem Einfluss seiner eigenen Dynamik (sogenannte Feedback- oder Rückkopplungseffekte), durch natürliche externe Einflüsse wie Änderungen in der Sonneneinstrahlung (Milanković-Zyklen) oder durch anthropogene Einflüsse wie die Verbrennung fossiler Brennstoffe. Die unterschiedlichen Teile des Klimasystems reagieren wiederum unterschiedlich schnell auf externe Antriebsfaktoren. Am schnellsten reagieren die Atmosphäre und der oberflächennahe Teil der Ozeane. In der Tiefsee dagegen verlaufen klimatische Veränderungen sehr langsam, die großen Eisschilde reagieren noch langsamer auf Veränderungen. Diese Liste von Systemelementen und Wechselwirkungen lässt sich beinahe beliebig fortsetzen, denn das Klimasystem ist hochkomplex mit unüberschaubar vielen Systemelementen. Es hat stochastische Züge, und ist von uns beeinflussbar, aber nicht steuerbar. In unserem CI wurde eine einfache Auffassung des Inhaltsbereichs *Klima als System* eingenommen, da vertiefte Betrachtungen systemischen Denkens (z. B. Rieß 2013) einen zusätzlichen, anderen Fokus des CIs bedingt hätten.

### Der Treibhauseffekt

Da der aktuelle Klimawandel auf menschliche Einflüsse zurückzuführen ist, stellt der Treibhauseffekt, insbesondere der anthropogene Anteil an diesem, einen weiteren zentralen Baustein einer Gesamtbetrachtung dar. Der Treibhauseffekt ist jedoch ein komplexes Phänomen, für dessen Verständnis mehrere Wissenselemente miteinander verknüpft werden müssen. Eine mögliche fachliche Klärung des Treibhauseffekts, die als Grundlage für eine geeignete Elementarisierung im Unterricht dienen kann, sieht so aus:

(Dunkle) Körper strahlen entsprechend ihrer Temperatur Energie ab. Das bezieht sich sowohl auf die Energiemenge als auch auf die Wellenlänge der Strahlung. Die Sonne strahlt überwiegend sichtbare Strahlung ab. Die Sonne ist Hauptenergielieferant für die Erde. Die Erde ist von einer Atmosphäre umgeben. Der Treibhauseffekt lässt sich in vier Schritten darstellen.

1. Schritt: Etwa ein Viertel der Sonnenstrahlung wird an den Wolken direkt in den Weltraum reflektiert; ein anderes Viertel wird von der Atmosphäre absorbiert. Ungefähr die Hälfte der Sonnenstrahlung gelangt ungehindert durch die Atmosphäre bis zum Erdboden. Helle Stellen wie Eis oder Schnee auf der Erdoberfläche reflektieren diese Strahlung unverändert zurück in den Weltraum.

2. Schritt: Dunkle Stellen wie Gestein oder Ozean absorbieren die Sonnenstrahlung und erwärmen sich. Die dunkle, erwärmte Erdoberfläche strahlt aufgrund ihrer Temperatur langwellige Wärmestrahlung ab. Das ist eine Strahlungsumwandlung von sichtbarer Sonnenstrahlung in Wärmestrahlung.

3. Schritt: Für die Wärmestrahlung ist die Atmosphäre auf Grund in Spuren vorkommender Gase (Wasserdampf, CO_2_ …) nur teilweise durchlässig. Die Atmosphäre wird von unten erwärmt. Das ist der natürliche Treibhauseffekt auf der Erde, der die mittlere Temperatur von −18 °C auf +15 °C Grad hebt. Es gibt einen zusätzlichen, vom Menschen verursachten Treibhauseffekt (auf +16 °C) durch erhöhten Eintrag von bspw. CO_2_ in die Atmosphäre.

4. Schritt: Die erwärmte Atmosphäre emittiert auch wieder langwellige Wärmestrahlung – auch nach unten. Dadurch wird die Erdoberfläche zusätzlich erwärmt. Im Endeffekt strahlt die Erde genauso viel Energie ab, wie von der Sonne eingestrahlt wird (Strahlungsgleichgewicht). Mit zunehmender CO_2_-Menge in der Atmosphäre wird auch zunehmend Wärmestrahlung von der Erdoberfläche von der Atmosphäre absorbiert. Dadurch verlässt weniger Energie die Erde (Strahlungsungleichgewicht). Mit steigender Temperatur steigt aber die Fähigkeit zur Abstrahlung von Energie. Es kommt zu einem neuen Strahlungsgleichgewicht bei erhöhter Temperatur der Erde.

### Der Kohlenstoffkreislauf

Anhand des Treibhauseffekts kann geklärt werden, warum es durch eine erhöhte Konzentration von Treibhausgasen in der Atmosphäre zu einem Anstieg der globalen Mitteltemperatur kommt. Offen bleibt dabei aber, wie es zu dieser Anreicherung an Treibhausgasen in der Atmosphäre kommen kann. Um diesen Anreicherungseffekt zu verstehen, braucht es ein basales Verständnis über den sogenannten Kohlenstoffkreislauf: Es bedarf also Wissen darüber, dass Kohlenstoff in unterschiedlichen Teilen oder Sphären des Klimasystems vorkommt, und dass sich dieser insbesondere zwischen der Atmosphäre, den Lebewesen und der Hydrosphäre bewegt (Speicher-Fluss-Schema), wobei CO_2_ selbst einen Speicher darstellt. Es gibt eine große Menge an Kohlenstoff, der auf natürliche Weise nicht maßgeblich an diesem Austausch beteiligt ist. Das ist etwa Kohlenstoff, der in Gesteinsform gebunden ist, sowie Kohlenstoff, der in Form von fossilen Brennstoffen (fest, flüssig und gasförmig) in der Erdkruste gelagert ist.

Ohne menschliche (oder externe natürliche) Einflüsse befindet sich der Austausch des Kohlenstoffs in einem natürlichen Gleichgewicht – es fließt also genauso viel Kohlenstoff von den Ozeanen und Lebewesen in die Atmosphäre, wie die Ozeane und Lebewesen aufnehmen. Dieses natürliche Gleichgewicht sorgte über Jahrhunderte für eine relativ konstante CO_2_-Konzentration in der Atmosphäre. Durch die Verbrennung fossiler Brennstoffe wird jedoch zusätzliches CO_2_ in die Atmosphäre eingebracht, wodurch der natürliche Kohlenstoffkreislauf gestört wird. Darüber hinaus stellt das Abholzen von Wald, früher im Rahmen der Industrialisierung und heutzutage im Amazonas, wegen verminderter Photosynthese einen verminderten Abfluss aus dem Speicher Atmosphäre dar, und die Brandrodung stellt wegen vermehrter Atmung einen verstärkten Zufluss *in* den Speicher Atmosphäre dar. Zumindest im Erdzeitalter Karbon, als Kohlenstoff in den fossilen Brennstoffen gespeichert wurde, muss ein natürliches Fließ*un*gleichgewicht geherrscht haben.

Die Beschreibung der abgedeckten Inhaltsbereiche liefert einen Baustein für die Definition des zu messenden Konstrukts. In den nächsten beiden Kapiteln legen wir offen, welchem Verständnis von Lernendenvorstellungen wir in der Entwicklung des CCCI-422 gefolgt sind und auf welche bereits bekannten Vorstellungen zu den naturwissenschaftlichen Grundlagen des Klimawandels in der Testentwicklung aufgebaut wurde, um den zweiten Baustein zu liefern.

## Verständnis von (alternativen) Lernendenvorstellungen

Unter Lernendenvorstellungen verstehen wir in diesem Artikel und dementsprechend auch für die Interpretation des CCCI-422 alle individuellen gedanklichen Prozesse, über die Lernende zu einem spezifischen Kontext, in diesem Fall zu den naturwissenschaftlichen Grundlagen des Klimawandels, verfügen können (Heeg et al. 2021). In Anlehnung an die Beschreibung bei Heeg et al. (2021) entscheiden wir für die Entwicklung des CCCI-422 dabei nicht, ob diese von Personen in Situationen erstmalig ad hoc oder wiederholt konstruiert werden und somit als gefestigt angesehen werden können. Hauptsächlich deshalb, weil es uns nicht möglich erscheint, anhand des hier beschriebenen CIs zwischen gefestigten und ad hoc konstruierten Vorstellungen zu unterscheiden. Stimmen diese Vorstellungen nicht mit aktuellen wissenschaftlichen Vorstellungen überein, so bezeichnen wir diese ebenso in Anlehnung an Heeg et al. als alternative Lernendenvorstellungen, da diese im Rahmen konstruktivistischer Lerntheorien vielmehr als Ressource für die Initiierung von Lernprozessen denn als reines Hindernis gesehen werden sollten (z. B. Baalmann et al. 2004; Krumphals et al. 2022; Larkin 2012). Durch die Interpretation der Testverhalten auf den CCCI-422 soll also sowohl die Diagnose von fachlich angemessenen Vorstellungen (angemessene Antwort) als auch die Diagnose von alternativen Lernendenvorstellungen (Alternativantworten) zu den oben beschriebenen Inhaltsbereichen ermöglicht werden. Die Anzahl an vertretenen fachlich angemessenen Lernendenvorstellungen ist wiederum als Indikator für das konzeptuelle Verständnis von Personen zu interpretieren. Welche alternativen Lernendenvorstellungen zu den naturwissenschaftlichen Grundlagen des Klimawandels bereits aus der Literatur bekannt sind, beschreiben wir im nächsten Kapitel.

## Vorstellungen Jugendlicher zu naturwissenschaftlichen Grundlagen des Klimawandels

Die Analyse der anzunehmenden Lernendenvorstellungen der Zielgruppe über den zu adressierenden Inhaltsbereich ist einer der zentralen Schritte in der Entwicklung von CIs. Im konkreten Fall wurden dementsprechend Studien zu Lernendenvorstellungen im Bereich der naturwissenschaftlichen Grundlagen des Klimawandels herangezogen, die wir in Grundzügen in diesem Kapitel darstellen.

Das Thema Klimawandel zeigt sich grundsätzlich für SchülerInnen aller Bildungsniveaus als fachlich herausfordernd (Akaygun und Adadan 2021) und ist mit vielschichtigen Lernendenvorstellungen verbunden, die sich in zwei grobe Bereiche unterteilen lassen: einerseits in Vorstellungen zu allgemeinen fachlichen Inhaltsbereichen wie z. B. grundlegenden Konzepten der Thermodynamik und andererseits in Vorstellungen zu naturwissenschaftlichen Phänomenen und Mechanismen, die durch den Kontext Klimawandel spezifiziert sind wie z. B. dem Treibhauseffekt (Gorr 2021). Letztere werden in diesem Abschnitt entlang in der Literatur dargestellter Themenkomplexe überblicksartig zusammengefasst.

Wie internationale (vgl. z. B. Überblicke in Bhattacharya et al. 2021; Varela et al. 2020) Untersuchungen, aber auch Forschungsergebnisse im deutschsprachigen Raum (Schubatzky und Haagen-Schützenhöfer 2021) zeigen, geht die Mehrheit der Jugendlichen davon aus, dass aktuell ein Klimawandel stattfindet, der anthropogene oder andere, etwa natürliche, Ursachen hat. Als Ursachen für den aktuellen Klimawandel ziehen SchülerInnen tendenziell verschiedene Phänomene, die überwiegend aus dem Kontext der Umweltverschmutzung stammen, heran. Dazu gehören z. B. die Zerstörung der Ozonschicht, Luftverschmutzung oder radioaktive Abfälle (Huxster et al. 2015; Punter et al. 2011; Schuler 2011; Shepardson et al. 2012; Varela et al. 2020). Dementsprechend werden neben wissenschaftlich ableitbaren Klimawandelfolgen wie Erderwärmung, Abschmelzen der Polkappen oder Anstieg der Meeresspiegel auch Auswirkungen wie Hautkrebs, Herzinfarkt oder ein unverhältnismäßig hoher Temperaturanstieg vermutet (Varela et al. 2020).

Naturwissenschaftliche Mechanismen, die dem Klimawandel zugrunde liegen, sind komplex, nicht linear und erfordern zumindest basales systemisches Verständnis. Das Konzept *Klima als System *ist für SchülerInnen etwa herausfordernd, da ein mangelndes Bewusstsein über Rückkopplungseffekte vorherrscht (Jarrett und Takacs 2020; Schuler 2011; Shepardson et al. 2014). Ähnliches gilt für Wetter- und Klimaphänomene, die mitunter vermischt werden (Lombardi und Sinatra 2012), u. a. weil ein fehlendes Verständnis des Konzepts von *deep times* (Dodick und Orion 2003; Tasquier et al. 2016) ein vertieftes Verständnis des Unterschieds zwischen Wetter und Klima erschwert (Lombardi und Sinatra 2012).

Neben prinzipiellen Fehlannahmen zum Aufbau der Atmosphäre (Henriques 2002) lassen sich auch vielfältige Vorstellungen zu Treibhausgasen identifizieren: Während CO_2_ typischerweise als Treibhausgas kategorisiert wird, trifft das für Wasserdampf, Methan, FCKW oder Stickoxide häufig nicht zu (Jarrett und Takacs 2020; Liarakou et al. 2011; Shepardson et al. 2009). Zudem wird der Anteil an Treibhausgasen oftmals überschätzt (Jarrett und Takacs 2020; Schubatzky et al. 2020). Treibhausgase werden aber auch mit Luftverschmutzung in Verbindung gebracht (Chang und Pascua 2016).

Grundsätzlich fällt es SchülerInnen schwer, zwischen natürlichem Treibhauseffekt und durch menschliche Aktivitäten verstärktem Treibhauseffekt zu unterschieden (Varela et al. 2020). Zu Mechanismen des Treibhauseffekts selbst sind zahlreiche Vorstellungen dokumentiert (z. B. Jakobsson et al. 2009; Jarrett und Takacs 2020; Keller 2006; Niebert und Gropengiesser 2013; Reinfried et al. 2010; Schuler 2011; Shepardson et al. 2014), die sich in Erwärmung durch mehr Einstrahlung und oder durch weniger Abstrahlung sowie in die Vermischung verschiedener beteiligter Strahlungsarten (UV, IR, sichtbares Licht) einteilen lassen. Erwärmung durch mehr Einstrahlung wird über die Ozonschicht zerstörende Mechanismen oder durch eine höhere Durchlässigkeit der Atmosphäre aufgrund des vorhandenen Ozonlochs erklärt (z. B. Niebert 2010). Erwärmung durch geringere Abstrahlung wird häufig über semipermeable Schichten (Ozonschicht oder alternativ Schicht aus hochkonzentrierten Treibhausgasen) erklärt, die die Re-emission einfallender Sonnenstrahlung durch Mehrfachreflexionen vermindert.

Als weitere Herausforderung im Verständnis des Klimawandels stellt sich auf Basis fachdidaktischer Forschung das Verständnis des Kohlenstoffkreislaufes heraus. Schwierigkeiten ergeben sich durch ein fehlendes Verständnis von Mechanismen der Bindung und der Freisetzung von Kohlenstoff bei Flüssen zwischen Kohlenstoff-Speichern (Bhattacharya et al. 2021; Düsing et al. 2019; Jarrett und Takacs 2020; Niebert 2010) wie durch eine Unterscheidung in „natürliches“ und „künstliches“ CO_2_ mit unterschiedlichen Entstehungsursprüngen.

Durch die Beschreibung der Inhaltsbereiche als auch potenzieller alternativer Lernendenvorstellungen sehen wir den Punkt der Beschreibung des zu messenden/erhebenden Konstrukts an dieser Stelle abgeschlossen. Für die intendierte Testwertinterpretation unseres CIs im Sinne der Personenfähigkeit dient dementsprechend folgende Definition:

Die durch den CCCI-422 ermittelte Personenfähigkeit steht für das konzeptuelle Verständnis der naturwissenschaftlichen Grundlagen des Klimawandels zu den Inhaltsbereichen *Atmosphäre unserer Erde, Der Unterschied zwischen Wetter und Klima, Klima als System, Kohlenstoffkreislauf *und* Treibhauseffekt. *In einer mehrdimensionalen Betrachtungsweise des CIs anhand dieser Inhaltsbereiche stehen die jeweiligen Personenfähigkeiten in den genannten Dimensionen für das jeweilige konzeptuelle Verständnis der naturwissenschaftlichen Grundlagen des Klimawandels in den genannten Dimensionen.

Bevor wir anschließend die Entwicklung des Testinstruments darstellen, muss noch der verfolgte Validitätsansatz für den CCCI-422 beschrieben werden, um den Zweck sowie den Nutzen der unterschiedlichen Entwicklungsschritte in den Gesamtzusammenhang der Entwicklung einordnen zu können.

## Validitätskonzept

Validierung kann laut den AERA-Richtlinien als „a process of constructing and evaluating arguments for and against the intended interpretation of test scores and their relevance to the proposed use“ (AERA 2014, S. 14) angesehen werden. Die Validität als solche wird in diesem Zusammenhang also nicht als eine einmalig festgestellte, nicht veränderbare Eigenschaft eines Testinstruments definiert, sondern wird vielmehr – dem argumentbasierten Validitätskonzept folgend – als Eigenschaft der intendierten Interpretation und Nutzungsszenarien des Testinstruments angesehen (Kane 2013). Dazu können sowohl empirische Evidenzen als auch theoretische Argumente herangezogen werden (Meinhardt et al. 2018). Das bedeutet wiederum, dass es keine standardisierten Verfahren zur Validierung von Testinstrumenten geben kann, die Argumente sollten aber dennoch auf konsistente theoretische Annahmen gestützt werden (AERA 2014; Berger et al. 2019; Meinhardt et al. 2018). Anhand des CCCI-422 soll es möglich sein, konzeptuelles Verständnis und Lernendenvorstellungen ab der achten Schulstufe zu diagnostizieren und Lernverläufe darstellen zu können. Um diese Interpretation zu ermöglichen, sehen wir es dementsprechend als notwendig an, folgende Arten von Argumenten für die valide Interpretation des Testverhaltens auf den CCCI-422 zu formulieren:

1. Es braucht Argumente für die inhaltliche Validität der Items. Darunter verstehen wir in Anlehnung an Berger et al. (2019) sowie Hartig et al. (2012), dass alle Items des CCCI-422 das zu untersuchende Konstrukt erheben und die Items möglichst keine Aspekte erheben, die für das Konstrukt keine Rolle spielen und zusätzlich die unterschiedlichen Inhaltsbereiche im Instrument weder über- noch unterrepräsentiert sind.
2. Im Sinne einer kognitiven Validität der zu diagnostizierenden Lernendenvorstellungen ist sicherzustellen, dass die Antworten der Items auch tatsächlich typischen Antwortmustern von Lernenden ab der achten Schulstufe entsprechen.
3. Um eine Interpretation der Gesamt-Personenfähigkeit als konzeptuelles Verständnis zu den naturwissenschaftlichen Grundlagen sowie der Personenfähigkeiten in den jeweiligen Inhaltsbereichen als konzeptuelles Verständnis in den jeweiligen Inhaltsbereichen zu ermöglichen, braucht es Argumente für die strukturelle Validität des Testinstruments sprechen. Es sollte sich also sowohl eine eindimensionale als auch fünfdimensionale Struktur (anhand der Inhaltsbereiche) empirisch wiederfinden lassen.
4. Um das konzeptuelle Verständnis über naturwissenschaftliche Grundlagen des Klimawandels unterschiedlicher Zielgruppen (etwa SchülerInnen, (angehender) Lehrkräfte oder ExpertInnen) zuverlässig deuten zu können, braucht es Argumente externer Validität. Externe Validität bezieht sich dabei auf den Vergleich der Testwerte mit externen Faktoren, die insgesamt ein Argument für Konstruktvalidität unterstützen. Darunter verstehen wir unter anderem den Zusammenhang der Testwerte mit einem von außen angelegten Kriterium wie etwa einer bestimmten Gruppenzugehörigkeit oder dem Zusammenhang mit einem theoretisch naheliegenden Konstrukt.
5. Um die Entwicklung von Lernverläufen darzustellen, aber auch um Interventionen zum konzeptuellen Verständnis der naturwissenschaftlichen Grundlagen des Klimawandels evaluieren zu können, braucht es Argumente für die Instruktionssensitivität des CIs in unterrichtlichen Settings. Anhand des CIs müssen dementsprechend Entwicklungen im konzeptuellen Verständnis der Lernenden abgebildet werden können.

Im nächsten Kapitel beschreiben wir das konkrete Vorgehen bei der Entwicklung des CCCI-422. Die unterschiedlichen Studien tragen dabei zu den soeben genannten Punkten bei. Deren Beitrag wird daher am Ende jedes Unterkapitels separat diskutiert, um eine abschließende Bewertung der Validität im Diskussionskapitel zu ermöglichen.

## Vorgehen bei der Testentwicklung

**Figure 1:**
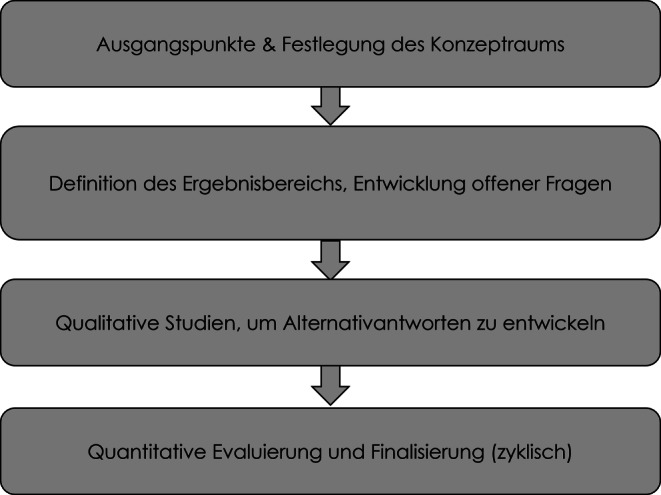

Ausgangspunkt für die Entwicklung des in diesem Artikel vorgestellten Concept Inventories (CCCI-422) war ein existierendes Climate Change Concept Inventory (CCCI) aus Australien (Jarrett und Takacs 2020), das übersetzt und in einer Vorstudie mit *N* = 338 SchülerInnen am Beginn der Sekundarstufe II (unterschiedliche Schultypen) in der Steiermark (Ö) erprobt wurde (Schubatzky et al. 2020). Das Instrument bestand aus 30 Multiple-Choice Fragen zu acht Inhaltsbereichen. Bei dieser Pilotierung fiel jedoch auf, dass einige Item-Formulierungen der Ursprungsversion verbesserungswürdig waren, zwei Items offensichtlich missverständlich formuliert waren, und das australische CCCI keine ausgewogene Item-Konzept-Verteilung aufwies. Nach dieser Pilot-Studie wurde deshalb entschieden, einen Klimawandel-Concept Inventory grundlegend neu zu entwickeln. Das Vorgehen orientierte sich dabei an der oben beschriebenen Vorgehensweise bzw. akzeptierten Richtlinien (AERA 2014; Berger et al. 2019; Meinhardt et al. 2018) und ist überblicksartig in Abb. 1 dargestellt.

Für die Entwicklung eines geeigneten Multiple-Choice Fragebogens wurden durch Sichtung bestehender Literatur sowie ExpertInneninterviews (*N* = 8 KlimawissenschaftlerInnen in Österreich und der Schweiz) fünf zentrale Inhaltsbereiche identifiziert, wie oben beschrieben und dargestellt:

- Atmosphäre unserer Erde,
- Treibhauseffekt,
- Klima als System,
- Unterschied Wetter – Klima,
- Kohlenstoffkreislauf.

### Entwicklung offener Antworten und Ableitung von Antwortalternativen

Der nächste Schritt bestand in der Entwicklung offener Fragen zu den jeweiligen Inhaltsbereichen. Daraus wurde für jeden der fünf Inhaltsbereiche ein Interviewleitfaden entwickelt und Interviews mit SchülerInnen und Studierenden wurden geführt. Dafür wurden 44 Interviews mit SchülerInnen der 8., 9. und 11. Klassenstufe durchgeführt. Aufgrund der COVID-19-Pandemie konnten weitere 8 SchülerInnen der 9. Klassenstufe nur auf Distanz mit Fragebögen mit offenem Antwortformat befragt werden. Zusätzlich wurden als Gelegenheitsstichprobe 8 Studierende vorwiegend nicht-naturwissenschaftlicher Fächer interviewt. Formulierungen der Itemstämme, Attraktoren und Distraktoren wurden literaturbasiert und anhand von diesen insgesamt über 50 Interviews in einem inhaltsanalytischen Vorgehen (Kuckartz 2016) entwickelt. Die Anzahl der Distraktoren wurde an dieser Stelle noch nicht eingeschränkt, weil a priori nicht absehbar war, welche der verwendeten Distraktoren sich in zukünftigen quantitativen Evaluierungen des CIs als bedeutungsvoll herausstellen würden. Dieses Vorgehen in der Itementwicklung betrachten wir als Argument für die kognitive Validität der Items, da alle Antworten typischen Antwortmustern von Lernenden entsprechen. Als Argument dagegen könnte angeführt werden, dass sich aufgrund einer vergleichsweise kleinen Stichprobe nicht auf typische Antwortverhalten schließen lässt, weshalb für die Zukunft weitere Studien zur kognitiven Validität der Interpretation der Antworten zu empfehlen sind, etwa durch lautes Denken während der Testbearbeitung (z. B. Große-Heilmann et al. 2022). Schließlich wurde eine Erstversion des CCCI-422 entwickelt, die aus insgesamt 40 Items mit jeweils einem Attraktor und jeweils zwischen drei und sieben Distraktoren bestand.

### Quantitative Pilotierungsstudien

Für die Finalisierung des in diesem Artikel vorgestellten Concept Inventories CCCI-422 wurden mehrere quantitative Pilotstudien durchgeführt, die in diesem Abschnitt dargestellt werden.

#### Studie zur rudimentären Itemauswahl

Die erste quantitative Pilotierung wurde mit insgesamt *N* = 10 Studierenden des Lehramts Physik sowie *N* = 23 Studierenden eines technischen Studiengangs durchgeführt. Das Ziel dieser Studie war es, die eingesetzten Items sowie den Gesamttest prinzipiell hinsichtlich der Eignung bei einer Gruppe, bei der wir zumindest ein rudimentäres Verständnis erwarteten, zu prüfen. Azizan et al. (2020) konnten aufzeigen, dass eine relativ stabile Messung von Item- und Personenreliabilitäten anhand eines Rasch-Modells bereits dann möglich ist, wenn die Anzahl der Items in etwa der Anzahl an administrierten Testpersonen entspricht, die Stichprobe sollte aber zumindest 30 Personen umfassen. Wir berichten deshalb bereits an dieser Stelle Testkennwerte in einem IRT-Framework, eine abschließende Bewertung der Fitwerte sowie der Personenreliabilität ist aufgrund der kleinen Stichprobe jedoch noch nicht zulässig. Es wurden einerseits Personenreliabilitätswerte berechnet sowie Punkt-Moment Korrelationen mit dem Gesamtscore (r > 0,2) bestimmt. Außerdem wurden Infit- und Outfit-Werte für die einzelnen Items unter Nutzung eines Rasch-Modells bestimmt, um Hinweise auf potenziell irreführend formulierte Items zu finden. Auf Basis dieser Analyse wurden insgesamt drei Items ausgeschlossen, die restlichen Items zeigten Infit- und Outfitwerte zwischen 0,8 und 1,2, die somit laut Bond und Fox (Bond und Fox 2015) im geeigneten Bereich für Infit- und Outfitwerte liegen. Nach Ausschluss dieser drei Items zeigte der Test eine WLE-Personenreliabilität von 0,82 für diese Stichprobe.

#### Reliabilitätsprüfung und Distraktorenauswahl

Die zweite quantitative Pilotierung wurde mit insgesamt *N* = 153 Personen durchgeführt. Unter diesen 153 Personen waren 92 SchülerInnen der 8. bis 12. Jahrgangsstufe aus Österreich und Deutschland sowie 61 Erstsemesterstudierende eines technischen Studiengangs in Deutschland. Ziel dieser zweiten Pilotierungsstudie war es, die überarbeitete und gekürzte Version des CCCI-422 unter Nutzung des Rasch-Modells hinsichtlich Itemfit sowie Personenreliabilität zu überprüfen, um zu einer möglichst finalen Version des Testinstruments zu gelangen. Die berechneten Infit- sowie Outfitwerte in dieser zweiten Pilotstudie bewegten sich alle im Bereich zwischen 0,75 < Infit/Outftit < 1,25 und sind somit als noch akzeptabel zu betrachten (Bond und Fox 2015). Die WLE-Personenreliabilität lag bei 0,71 und ist als ebenso ausreichend zu betrachten (Boone et al. 2014).

#### Studie zur externen Validität

**Figure 2:**
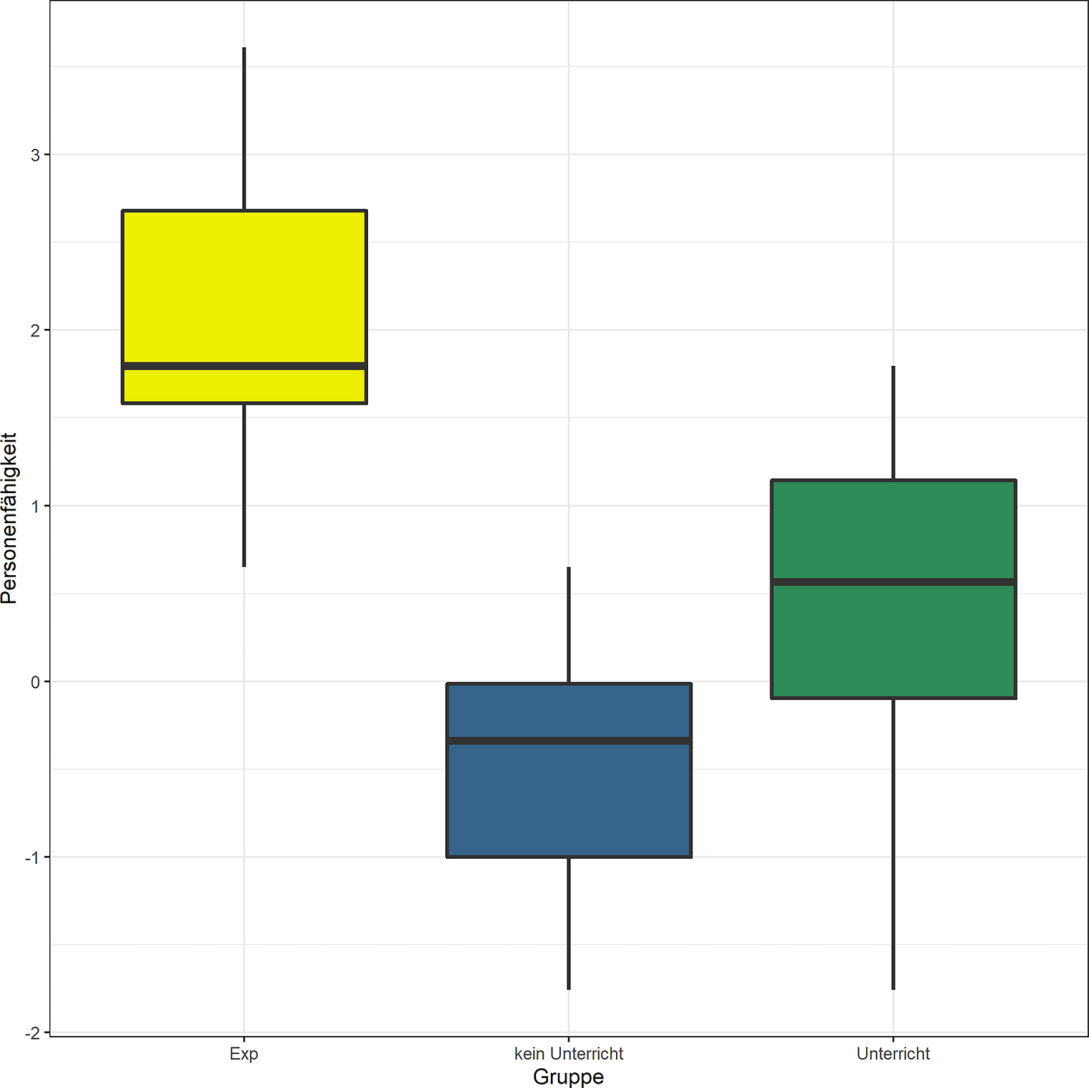

Die dritte quantitative Pilotierung wurde mit insgesamt *N* = 58 SchülerInnen der 11. Jahrgangsstufe aus Österreich und Deutschland sowie *N* = 7 aktiven Scientists for Future durchgeführt. Die SchülerInnen teilten sich dabei in zwei Gruppen auf: 18 SchülerInnen waren noch nicht zum Thema Klimawandel unterrichtet worden, die restlichen SchülerInnen hatten erst kürzlich die naturwissenschaftlichen Grundlagen des Klimawandels im Physikunterricht behandelt. Das Ziel dieser dritten Pilotierung bestand also insbesondere darin, Argumente für die externe Validität anhand der Methode der bekannten Gruppen zu generieren, um in zukünftigen Einsätzen zuverlässig unterschiedliche Wissensstände diagnostizieren zu können. Die Hypothese war demnach, dass die Scientists for Future im Mittel einen signifikant höheren Score erreichen würden als SchülerInnen, die bereits zum Thema unterrichtet wurden, welche im Mittel wiederum einen höheren Score erreichen würden als die SchülerInnen, die bisher noch keinen Physikunterricht zum Thema Klimawandel hatten. Diese Hypothese wurde anhand einer ANOVA sowie TukeyHSD-Tests überprüft, in Abb. 2 ist ein Boxplot der Scores der jeweiligen Gruppen dargestellt. Vor der Analyse wurde ein Levene’s Test zur Überprüfung der Varianzhomogenität durchgeführt, die bestätigt werden konnte (*p* = 0,481). Die Analyse zeigte, dass sich die Gruppen signifikant unterscheiden, (F(62,2) = 40,4, *p* < 0,001), mit einer *Effektstärke* von $\eta ^{2}=0{,}55$. Für anschließende paarweise Vergleiche wurden TukeyHSD-Tests sowie Cohen’s d als Effektstärke hinzugezogen. Es unterscheiden sich dabei alle drei Gruppen laut TukeyHSD-Tests signifikant, sowohl die Gruppen kein Unterricht und Unterricht (*p* < 0,001, *d* *=* *1,31*), als auch die Gruppen Exp und Unterricht (*p* < 0,001, *d* *=* *1,76*) und die Gruppen Exp und kein Unterricht (*p* < 0,001, *d* *=* *3,74*). Als Limitation muss angeführt werden, dass sich die Stichproben der unterschiedlichen Gruppen hinsichtlich ihrer Gruppengröße unterschieden. Außerdem wurden die kognitiven Fähigkeiten sowie die Lesekompetenz der ProbandInnen nicht erhoben, sodass hier eine Vergleichbarkeit nicht restlos sichergestellt werden kann. Zumindest wurde anhand des Regensburger Analysetools für Texte (Wild und Pissarek, o.J.) sichergestellt, dass die Items der durchschnittlichen Lesekompetenz einer/s Lernenden am Ende der 8. Schulstufe entsprechen. Trotz dieser Limitation sehen wir dieses Ergebnis als externes Validitätsargument, da sich die vermuteten Unterschiede im Wissensstand der ProbandInnen auch in den Daten widerspiegeln.

#### Studien zur Instruktionssensitivität.

Die vierte Studie diente zur Generierung von Hinweisen für die Instruktionssensititivät des hier vorgestellten CCCI-422. Dazu wurde der CI in einer österreichischen Schulklasse der 11. Schulstufe mit *N* = 21 Schülerinnen und Schülern vor sowie nach einer Instruktion zum Thema Klimawandel eingesetzt. Das Ziel war dabei zu untersuchen, ob es mit dem CCCI-422 prinzipiell möglich ist, auch für die aktuelle Unterrichtspraxis typische Lernzuwächse abzubilden, um so zusätzlich die ökologische Validität dieser Pilotierungsstudie zu erhöhen. Dabei zeigte sich anhand eines t‑Tests insgesamt ein signifikanter Zuwachs der mittleren Personenfähigkeit (t(20) = 3,72, *p* < 0,01) mit einer Effektstärke von *d* *=* *0,81*. In einer weiteren Erhebung wurde der Wissenszuwachs von *N* = 8 Lehramtsstudierenden in einem Vorbereitungsseminar auf das Praxissemester untersucht in dem der Fokus auf dem Thema Klimawandel lag, wobei sich auch hier ein Wissenszuwachs messen ließ (mit einer Effektstärke von *d* *=* *0,90*), nähere Informationen zur Statistik sind in (Nordmeier und Tabrizi im Druck) zu finden. Die dritte Erhebung wurde mit Studierenden des Bauingenieurwesens in Deutschland durchgeführt. Im Rahmen einer Vorlesung für Physik wurden wöchentlich Klimawandelfakten in Anlehnung an den CCCI-422 präsentiert, außerdem wurde an einem Termin der natürliche und der menschengemachte Treibhauseffekt behandelt. Bei einer Stichprobe von *N* = 59 Studierenden zum Beginn und *N* = 21 Studierenden zum Ende des Semesters stieg der Mittelwert richtig beantworteter Fragen statistisch signifikant (t(20) = 6,19, *p* < 0,001) von 16,6 auf 22,1 mit einer Effektstärke von *d* *=* *1,35* an.

Zusammengefasst sehen wir die Ergebnisse dieser drei Erhebungen als Hinweise für die Instruktionssensitivität des CCCI-422 bei unterschiedlichen Zielgruppen. Als Limitation können auch hier die fehlende Erhebung der Lesekompetenz der ProbandInnen und die Auswahl der Stichprobe im Sinne von Gelegenheitsstichproben genannt werden.

Da sich in den letzten drei Pilotierungsstudien kein weiterer Bedarf für die Überarbeitung von Items zeigte, wurde an dieser Stelle der Entschluss gefasst, eine abschließende Erhebung mit einer möglichst großen Stichprobe, zusammengesetzt aus unterschiedlichen Populationen, durchzuführen. Nicht zuletzt sollte damit ein belastbares Argument für Konstruktvalidität der intendierten Interpretation überprüft werden.

## Hauptstudie und Überprüfung des finalen Testinstruments

### Beschreibung der Itemauswahl für das finale Testinstrument

Der finale CI besteht aus insgesamt 35 Items, die sich auf die fünf bereits beschriebenen Inhaltsbereiche aufteilen. Die Items sind im Single-Choice Format formuliert, mit jeweils einer richtigen Antwort und drei bis fünf Distraktoren. Die Anzahl der Distraktoren unterscheidet sich aufgrund der in den Pilotstudien durchgeführten Distraktorenanalysen, sodass nur noch Alternativantworten herangezogen wurden, die in den Pilotierungsstudien von zumindest 5 % des Samples gewählt wurden. Es gab ein Item, bei dem die korrekte Antwort in weniger als 5 % der Fälle gewählt wurde, dieses wurde dennoch weiterhin beibehalten. Zudem wurden die Items post-hoc anhand von Formulierungsguidelines für Multiple-Choice Items abschließend überprüft (Haladyna et al. 2002).

**Figure 3:**
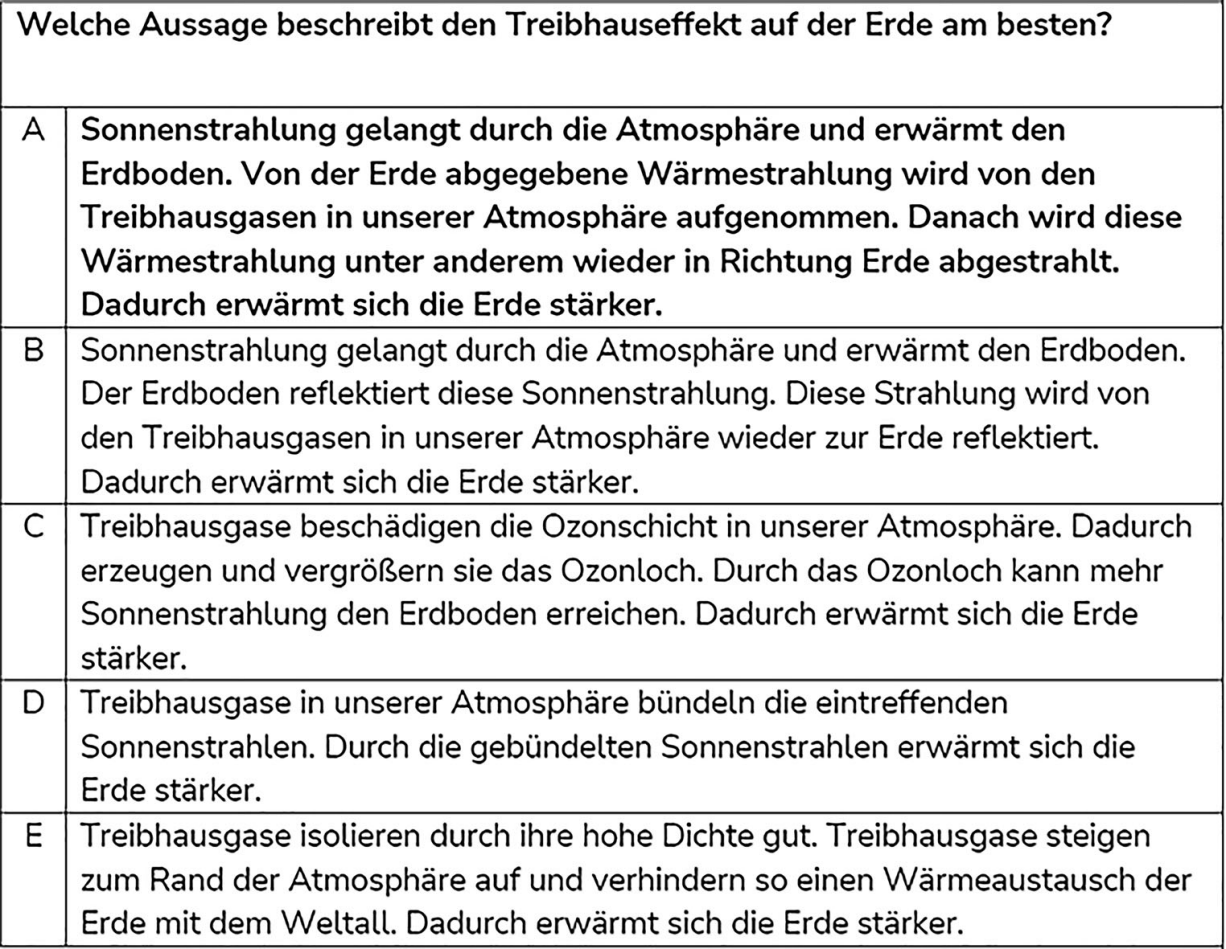

**Figure 4:**
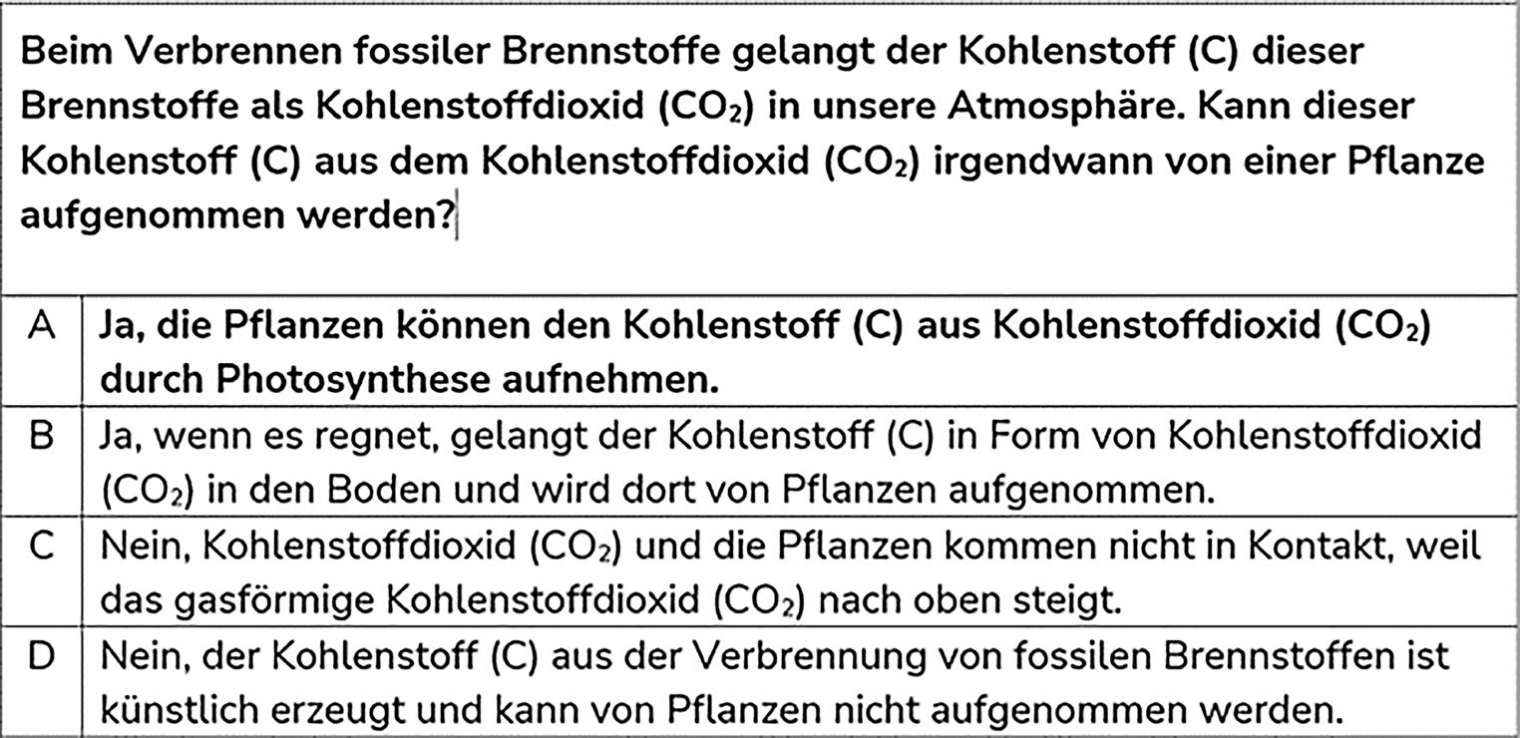

Die Items fragen überwiegend konkrete Vorstellungen ab, wie etwa jene zum *Treibhauseffekt *(siehe Abb. 3) oder zum *Kohlenstoffkreislauf *(siehe Abb. 4). Insbesondere zum Inhaltsbereich *Atmosphäre unserer Erde* gibt es jedoch auch Items, die einzelne Fakten abfragen, wie etwa den Anteil an Treibhausgasen in der Atmosphäre. Diese Items wurden aufgenommen, weil in bisheriger Literatur häufig Missverständnisse über die Zusammensetzung der Atmosphäre genannt wurden.

Der maximale klassische Testscore beläuft sich somit auf 35 Punkte bei 35 dichotomen Items, wobei die Analyse von Alternativantworten auf einzelne Items die Identifikation von Missverständnissen oder Vorstellungen ermöglicht. Das gesamte Testinstrument ist im Anhang zu finden und unter einer CC-BY-SA 4.0-Lizenz veröffentlicht. Die finale Überprüfung des Testinstruments wurde unter Nutzung des Rasch-Modells anhand probabilistischer Testtheorie durchgeführt.

### Stichprobe

Die finale Stichprobe besteht aus *N* = 783 SchülerInnen und *N* = 48 Physik-Lehramtsstudierenden aus Deutschland und Österreich, die durchschnittlich im 5. Fachsemester (4,89 ± 3,75) waren (siehe Tab. 1 für SchülerInnen, Tab. 2 für Studierende). Der CI wurde inklusive zusätzlicher Fragen demographischer Natur in Limesurvey (Engard 2009) als Online-Test umgesetzt, der auf PC oder Smartphone bearbeitet werden kann. Die Erhebung mit SchülerInnen wurde online, jedoch in Schulklassen während regulären Schulunterrichts durchgeführt. Die Erhebung mit Studierenden wurde ebenso online in Seminaren oder Lehrveranstaltungen an Universitäten durchgeführt. Die Teilnahme an der Studie war freiwillig. Die durchschnittliche Bearbeitungsdauer belief sich auf 17,2 min, die Standardabweichung auf 5,9 min. Für das finale Datenset wurden alle Bearbeitungen, die unter 5,4 min (Mittelwert minus doppelter Standardabweichung) lagen, ausgeschlossen, weil wir davon ausgehen, dass eine Bearbeitung in geringerer Zeit nicht ernsthaft gewährleistet werden kann.

**Table 1:** 

Merkmal	*n*	%
*Bundesland/Staat*		
Nordrhein-Westfalen/Deutschland	503	68
Österreich	232	32
*Klassenstufe*		
Klassenstufe 8	59	8
Klassenstufe 9	50	7
Klassenstufe 10	19	3
Klassenstufe 11	560	76
Klassenstufe 12	47	6
*Einschätzung, ob aktuell ein Klimawandel stattfindet*		
Ja	693	94
Nein	9	1
Unsicher	33	4
*Einschätzung, ob der aktuelle Klimawandel menschenverursacht ist*		
Hauptsächlich menschlicher Ursache	515	70
Hauptsächlich natürlicher Ursache	10	1
Gleichermaßen menschlicher und natürlicher Ursachen	196	27
Unsicher, wovon der Klimawandel verursacht wird	14	2

**Table 2:** 

Merkmal	*n*	%
*Bundesland/Staat*		
Berlin	9	19
Österreich	19	40
Nordrhein-Westfalen	20	42
*Einschätzung, ob aktuell ein Klimawandel stattfindet*		
Ja	47	98
Nein	0	0
Unsicher	1	2
*Einschätzung, ob der aktuelle Klimawandel menschenverursacht ist*		
Hauptsächlich menschlicher Ursache	39	81
Hauptsächlich natürlicher Ursache	1	2
Gleichermaßen menschlicher und natürlicher Ursachen	8	17
Unsicher, wovon der Klimawandel verursacht wird	0	0

### Ergebnisse der Rasch-Modellierung

**Figure 5:**
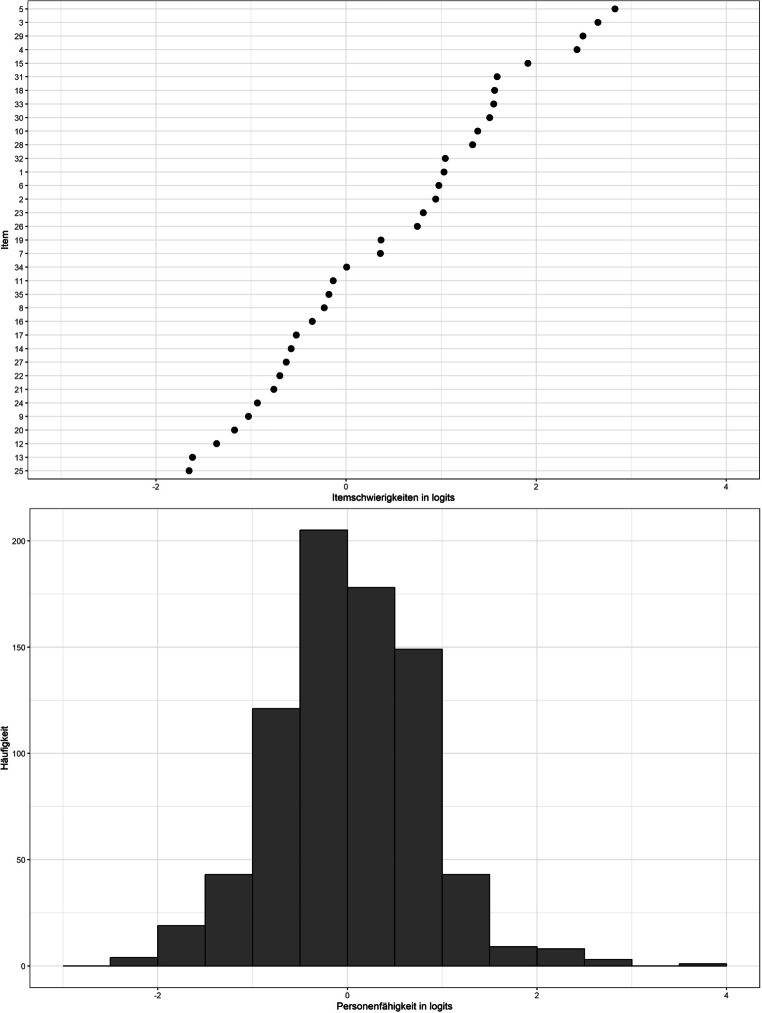

In diesem Kapitel werden die wesentlichen Kennwerte und Ergebnisse der Rasch-Modellierung dargestellt. Die WLE-Personenreliabilität beläuft sich dabei auf 0,72 für die Gesamtstichprobe, auf 0,65, wenn nur die Stichprobe der SchülerInnen für die Schätzung herangezogen wird, und auf 0,81, wenn nur die Stichprobe der Studierenden herangezogen wird. Eine Differential Item Functioning (DIF-) Analyse (SchülerInnen und Studierende als Gruppe) anhand der ETS-Klassifizierung (Zwick 2012) zeigt, dass 34 der 35 Items eine Klassifizierung als A oder B aufweisen und somit von keinem oder geringem DIF zeugen, lediglich ein Item (Frage zum Anteil der Treibhausgase in der Atmosphäre) kann als C‑Item eingeordnet werden und bevorteilt Studierende. Die Item-Reliabilität beträgt 0,99. Die Outfit-Werte der Items befinden sich im Bereich zwischen 0,8 und 1,43, die Infit-Werte im Bereich zwischen 0,9 und 1,1. Die vollständige Tabelle mit allen Fit-Werten befindet sich im Anhang. Die Itemschwierigkeiten decken einen Bereich zwischen −1,65 und 2,83 ab, die Personenfähigkeiten befinden sich im Bereich zwischen −2,36 und 3,71. Die Verteilungen dieser sind in der Wright-Map in Abb. 5 dargestellt.

**Figure 6:**
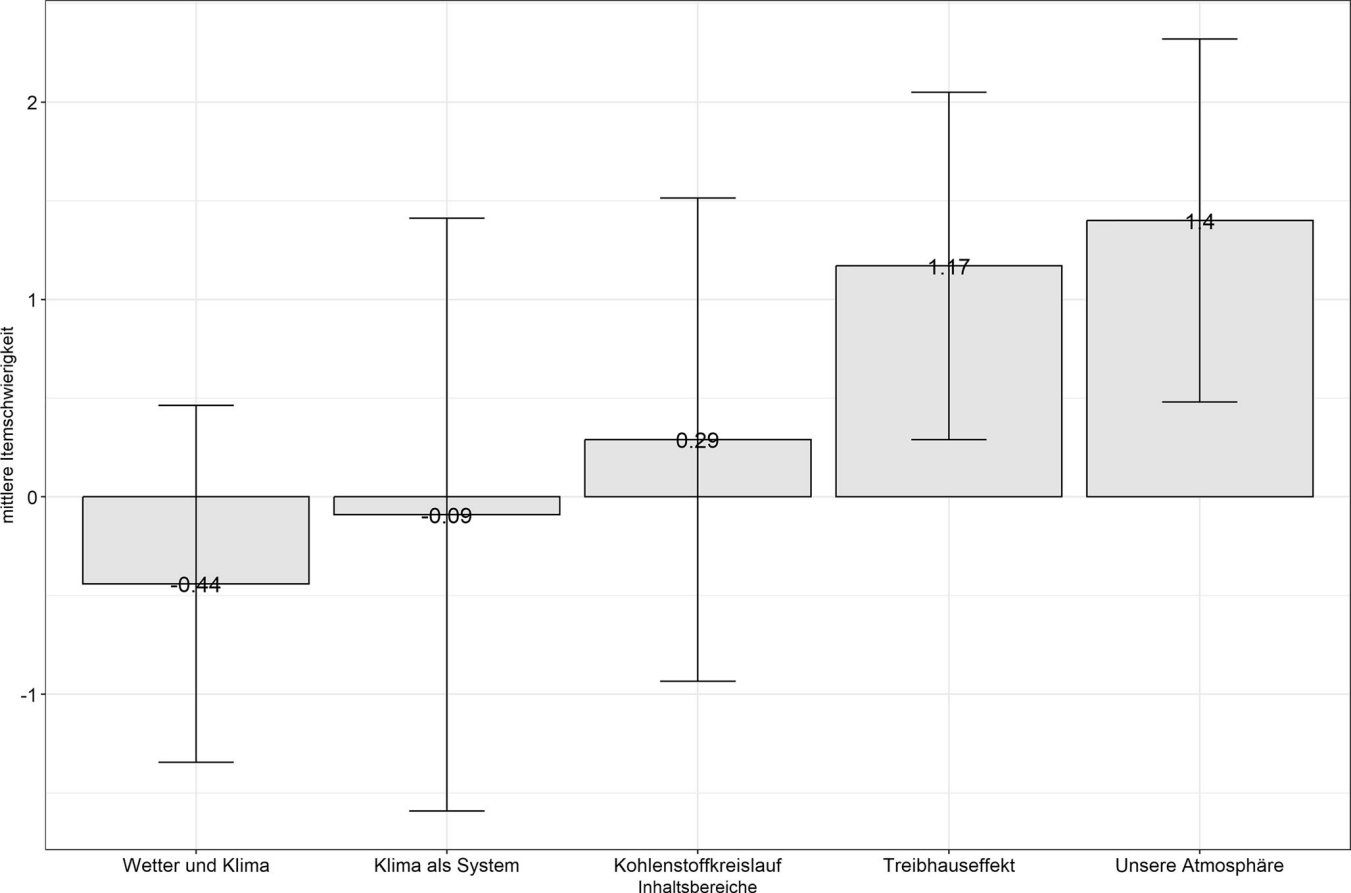

**Figure 7:**
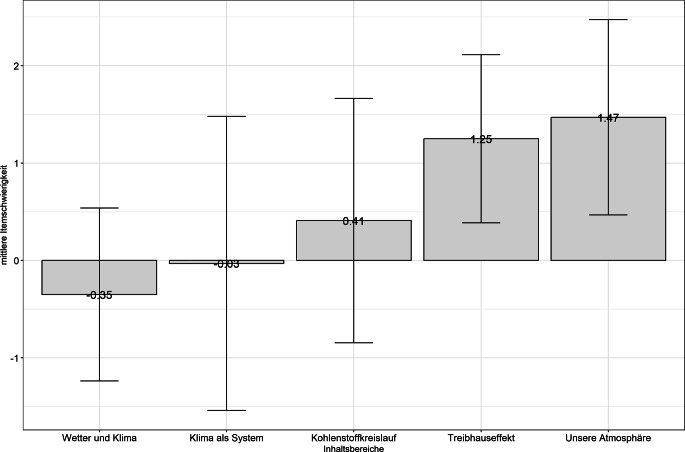

**Figure 8:**
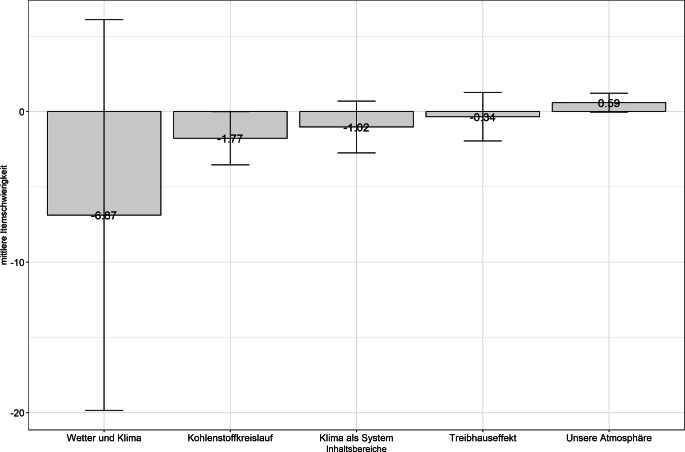

Auf Basis der geschätzten Itemschwierigkeiten können nun auch die durchschnittlichen Schwierigkeiten der fünf Inhaltsbereiche berechnet und dargestellt werden. Dazu wird die mittlere Itemschwierigkeit aller zu einem Inhaltsbereich gehörenden Items berechnet. Diese sind in Abb. 6 für die Gesamtstichprobe, für SchülerInnen in Abb. 7 und für Studierende in Abb. 8 dargestellt. Die Interpretation von Abb. 8 sollte besonders vorsichtig geschehen, da es sich um eine vergleichsweise kleine Stichprobe von 48 Studierenden im Lehramt Physik handelt. Die Fehlerbalken in den Abbildungen stellen die Standardabweichung der Itemschwierigkeiten pro Inhaltsbereich dar.

### Dimensionalität als Argument für Konstruktvalidität des Testinstruments

Als abschließender Baustein wurde versucht, durch die Durchführung einer mehrdimensionalen Raschanalyse, unter Nutzung des Pakets TAM, ein Argument für die Konstruktvalidität zu formulieren. Dazu wurden ein eindimensionales Modell sowie ein fünfdimensionales Modell entsprechend den dargestellten Inhaltsbereichen spezifiziert. Ein fünfdimensionales Raschmodell liefert somit insgesamt fünf unterschiedliche Personenfähigkeiten – je eine pro Inhaltsbereich (interpretierbar als konzeptuelles Verständnis des jeweiligen Inhaltsbereichs). Die Personenreliabilitäten für die jeweiligen Subskalen zu den Inhaltsbereichen sind in Tab. 3 dargestellt.

**Table 3:** 

Skala/Inhaltsbereich	Anzahl Items	EAP-Personenreliabilität	WLE-Personenreliabilität
Atmosphäre unserer Erde	6	0,56	0,34
Klima als System	8	0,74	0,52
Kohlenstoffkreislauf	6	0,78	0,63
Unterschied Wetter – Klima	7	0,77	0,54
Treibhauseffekt	8	0,72	0,62

Um Hinweise auf Zusammenhänge zwischen den einzelnen Inhaltsbereichen abzuleiten, wurden die Korrelationen zwischen den Personenfähigkeiten der jeweiligen Inhaltsfelder bestimmt und in Tab. 4 dargestellt.

**Table 4:** 

	Atmosphäre unserer Erde	Klima als System	Kohlenstoff-kreislauf	Unterschied Wetter – Klima	Treibhauseffekt
Atmosphäre unserer Erde	1	0,51	0,61	0,64	0,68
Klima als System	0,51	1	0,88	0,93	0,78
Kohlenstoff-kreislauf	0,61	0,88	1	0,88	0,88
Unterschied Wetter – Klima	0,64	0,93	0,88	1	0,81
Treibhauseffekt	0,68	0,78	0,88	0,81	1

**Figure 9:**
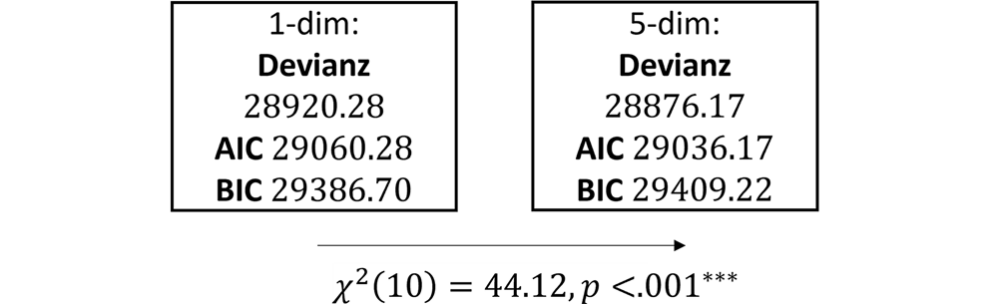

Als letzter Schritt wurde zudem ein Modellvergleich zwischen dem eindimensionalen und dem fünfdimensionalen Modell durchgeführt und in Abb. 9 dargestellt.

So zeigt sich, dass das fünfdimensionale Modell im Chi-Quadrat-Vergleich einen signifikant besseren Modellfit darstellt. Wirft man jedoch einen Blick auf das Bayes’sche Informationskriterium BIC, so zeigt sich ein niedrigerer Wert für das eindimensionale Modell.

## Interpretation der Hauptstudie

Bevor in der Diskussion ein Gesamtresümee über gesammelte Validitätsargumente, Limitation des Testinstruments sowie zukünftige, intendierte Einsatzzwecke und -szenarien gezogen wird, findet in diesem Kapitel eine Bewertung der Ergebnisse der abschließenden Haupterhebung statt.

### Bewertung der Personenreliabilitäten und Itemfit

Die ermittelten Personenreliabilitäten der Schätzung in einem eindimensionalen Modell für die unterschiedlichen Personengruppen (zwischen 0,65 und 0,81) können zumindest als zufriedenstellend bewertet werden und liegen in einem vergleichbaren Bereich zu bereits existierenden Konzepttests (z. B. Berger et al. 2019; Ivanjek et al. 2021). Wir vermuten, dass die geringere Reliabilität von 0,65 bei der Stichprobe der SchülerInnen auf fehlendes konzeptionelles Verständnis zurückzuführen ist, dieser Aspekt wird tiefer in der Diskussion im nächsten Kapitel aufgegriffen.

Ebenso sind die Personenreliabilitäten zu den Inhaltsbereichen *Klima als System, Kohlenstoffkreislauf, Unterschied Wetter – Klima* und *Treibhauseffekt* als zufriedenstellend zu betrachten. Die Personenreliabilität für den Inhaltsbereich *Atmosphäre unserer Erde *zeigt mit 0,56 einen höchstens akzeptablen Wert, der sich aus unserer Sicht auf die Faktenfrage zum Anteil der Treibhausgase in der Atmosphäre zurückführen lässt. Zu beachten ist jedoch, dass Schätzungen der Personenreliabilitäten anhand eines Weighted-Likelihood (WLE) Schätzers deutlich geringer ausfallen, sodass dies in der Betrachtung des fünfdimensionalen Modells jedenfalls zu berücksichtigen ist. Insgesamt sind diese Werte jedoch vor allem vor dem Hintergrund der jeweils geringen Itemanzahl (sechs bis acht Items) als zufriedenstellend zu betrachten.

Die Reliabilitäten können somit sowohl in einer Gesamtbetrachtung als auch in einer getrennten Schätzung der Schüler- und Studierendenpopulationen und in einer mehrdimensionalen Betrachtung als zumindest geeignet angesehen werden.

Die Itemfitwerte zeigen für 31 Items einen gut geeigneten Wert zwischen 0,8 und 1,2 und für vier Items zumindest einen akzeptablen Wert (Bond und Fox 2015; Boone et al. 2014) zwischen 0,8 und 1,43. Für diese vier Fälle lassen sich inhaltliche Begründungen für die erhöhten Outfitwerte formulieren. Für den Inhaltsbereich *Atmosphäre unserer Erde* etwa zeigt die Frage einen erhöhten Wert, in der nach dem Anteil an Treibhausgasen in unserer Atmosphäre gefragt wird. Diese Frage erwies sich als eine der schwierigsten Fragen im CCCI-422. Hier liegt die Vermutung nahe, dass es einige Personen gab, die zwar insgesamt kein ausgeprägtes Wissen über die naturwissenschaftlichen Grundlagen aufwiesen, aber diesen einen konkreten Fakt (Anteil der Treibhausgase) wussten oder gut geraten haben, wodurch der erhöhte Outfit-Wert zustande kommt. Dieser Umstand könnte aus unserer Sicht auch die C‑Klassifizierung dieses Items im Zuge der DIF-Analyse erklären. Eine ähnliche Begründung lässt sich auch für ein Item zum Treibhauseffekt mit dem Outfit-Wert von 1,4 formulieren: In diesem Item wird nach der mittleren Temperaturerhöhung der Erde durch menschliche Aktivitäten gefragt. Auch hier liegt die Vermutung nahe, dass Personen, die potenziell kein ausgeprägtes Wissen über die naturwissenschaftlichen Grundlagen des Klimawandels aufweisen, dennoch wissen, dass die Temperaturerhöhung zwischen 1 und 2 °Celsius beträgt, was zum erhöhten Outfit-Wert führen kann. Da diese Wissensfacetten jedoch sowohl in der Literatur als auch in den ExpertInneninterviews als wichtig betrachtet werden, wurden diese Items trotz nur akzeptablen Outfit-Werten im Testinstrument belassen.

### Bewertung des Konstruktvaliditäts-Arguments

Auf Basis der Analysen erscheint es an erster Stelle auch aus empirischer Sicht als möglich und sinnvoll, von einem eindimensionalen Konstrukt zu sprechen. Wir argumentieren damit also, dass sich die Personenfähigkeiten als konzeptuelles Verständnis der naturwissenschaftlichen Grundlagen des Klimawandels interpretieren lassen. In einem direkten Vergleich durch einen Chi-Quadrat Test stellt sich ein fünfdimensionales Modell anhand der identifizierten Inhaltsbereiche sogar als bessere Passung zu den Daten dar. Vergleicht man jedoch die Werte der beiden Modelle für das Bayes’sche Informationskriterium (BIC), welche strengere Strafen für das Erhöhen der Modellkomplexität miteinbezieht, so zeigt sich für das eindimensionale Modell ein niedrigerer BIC-Wert. Wir argumentieren dementsprechend also weiter, dass es auch möglich ist, Personenfähigkeiten zu den jeweiligen Inhaltsbereichen zu interpretieren. Wir lesen dieses Ergebnis derart, dass sich eine gewisse Modelläquivalenz zwischen dem eindimensionalen und fünfdimensionalen Modell zeigt, sodass der CCCI-422 anhand beider Modellspezifikationen eingesetzt werden kann (Burnham und Anderson 2004).

Die Entscheidung, welches der beiden Modelle (eindimensional oder fünfdimensional) in zukünftigen Studien eingesetzt werden sollte, hängt also im Wesentlichen vom intendierten Einsatzzweck ab. Die eindimensionale Personenfähigkeit stellt einen Indikator für das konzeptuelle Verständnis über naturwissenschaftliche Grundlagen dar und kann etwa in Studien genutzt werden, welche die Beziehung des konzeptuellen Verständnisses zu naturwissenschaftlichen Grundlagen des Klimawandels zu weiteren Variablen untersuchen. Der Einsatz des fünfdimensionalen Modells lässt hingegen einen differentiellen Schluss in Bezug auf die unterschiedlichen Inhaltsbereiche zu, wie anhand der Verteilungen der Itemschwierigkeiten in Abb. 6, 7 und 8 auch angedeutet wird, sodass dieses etwa in Interventionsstudien zu naturwissenschaftlichen Grundlagen des Klimawandels eingesetzt werden kann, um Lernverläufe zu den unterschiedlichen Inhaltsbereichen nachzuzeichnen.

## Diskussion

In dieser abschließenden Diskussion bewerten wir zunächst die von uns im Abschnitt Validitätskonzept formulierten Argumente für eine valide Interpretation der Personenfähigkeiten. Danach greifen wir einige noch bestehende Limitationen der Interpretation der Ergebnisse des CCCI-422 auf, bevor wir einen Ausblick auf potenzielle zukünftige Studien geben, die anhand des CCCI-422 durchgeführt werden könnten und sollten. Im Kapitel Validitätskonzept haben wir insgesamt fünf Argumente besprochen, die wir anhand der Teilstudien stützen möchten:

1. Es braucht Argumente für die inhaltliche Validität der Items. Alle Items wurden auf Basis der Beschreibungen der Inhaltsbereiche erstellt. Zudem zeigte sich in der Haupterhebung, dass alle Items dasselbe Konstrukt erheben. Wir argumentieren deshalb dafür, dass die Personenfähigkeit tatsächlich als konzeptuelles Verständnis über die naturwissenschaftlichen Grundlagen des Klimawandels interpretiert werden kann. Zu berücksichtigen ist jedoch nach wie vor, dass der CI aus einer physikdidaktischen Perspektive heraus entwickelt wurde und damit wohl auch einen physikalischen Schwerpunkt aufweist.
2. Im Sinne einer kognitiven Validität der zu diagnostizierenden Lernendenvorstellungen ist sicherzustellen, dass die Antworten der Items auch tatsächlich typischen Antwortmustern von Lernenden ab der achten Schulstufe entsprechen. Alle Antworten, sowohl Attraktoren als auch Distraktoren, beruhen zudem auf bekannten und/oder in unseren Interviews geäußerten Lernendenvorstellungen. Wir sehen darin ein Argument für die kognitive Validität, welches jedoch zukünftig noch ausgebaut werden könnte, etwa durch Lautes-Denken Studien anhand des finalen CIs.
3. In der Hauptstudie fanden wir sowohl eine eindimensionale als auch eine fünfdimensionale Struktur des Testinstruments. Wir argumentieren daher, dass die Gesamt-Personenfähigkeit als konzeptuelles Verständnis zu den naturwissenschaftlichen Grundlagen des Klimawandels sowie die Personenfähigkeiten in den jeweiligen Inhaltsbereichen als konzeptuelles Verständnis in den jeweiligen Inhaltsbereichen interpretiert werden können.
4. Im Abschnitt *Studie zur externen Validität* konnten wir signifikant unterschiedliche Testwerte für SchülerInnen ohne Instruktion, mit Instruktion und Scientists for Future finden, die auch der theoretischen Annahme entsprachen. In der Hauptstudie fanden wir zudem signifikant bessere Testwerte für die Gruppe der Studierenden als für die Gruppe der SchülerInnen. Wir argumentieren daher, dass der CCCI-422 das konzeptuelle Verständnis unterschiedlicher Zielgruppen erheben kann.
5. Im Abschnitt *Studien zur Instruktionssensitivität* konnten wir Lernerfolge unterschiedlicher Zielgruppen (SchülerInnen, Lehramtsstudierende, Studierende eines technischen Faches) abbilden und argumentieren daher, dass der CCCI-422 in zukünftigen Interventionsstudien in Unterrichtssettings eingesetzt werden kann.

Auf Basis dieser Argumente kommen wir zum Schluss, dass sich anhand des CCCI-422 das konzeptuelle Verständnis über die naturwissenschaftlichen Grundlagen des Klimawandels (in den erhobenen Inhaltsbereichen) ausreichend valide und reliabel messen lässt, sodass dieser in zukünftigen Studien zu den genannten Zwecken eingesetzt werden kann.

Trotz dieser Bewertung bleiben einige Punkte offen, die einerseits Limitationen der Interpretation der Ergebnisse des CCCI-422 darstellen, andererseits aber auch Anlass für zukünftige, weitere Untersuchungen sein können. So gibt etwa die Mischung aus Fragen zu eindeutig deklarativem Faktenwissen (wie dem Anteil der Treibhausgase in der Atmosphäre) und konzeptuellem Wissen Anlass zur Diskussion, denn folgt man der Definition von Concept Inventories streng, dürften derartige Faktenfragen nicht Teil des Testinstruments sein. Wir haben diese Faktenfragen dennoch integriert, weil sowohl Literatur als auch die ExpertInnen in Interviews diese Fakten als besonders bedeutsam für ein konzeptionelles Verständnis des Klimawandels herausstreichen. So könnte das Faktenwissen über den Anteil von Treibhausgasen in der Atmosphäre etwa auch stellvertretend für die irreführende Vorstellung stehen, dass eine große Wirkung (wie die Auswirkungen des Klimawandels) immer auch auf eine große Ursache (hoher Anteil an Treibhausgasen in der Atmosphäre) zurückzuführen sein muss. Zukünftige Studien sollten jedenfalls in den Blick nehmen, welche Rolle dieses Faktenwissen tatsächlich für das konzeptionelle Verständnis des Klimawandels darstellt, oder ob es sinnvoller wäre, etwa die Items zu *Atmosphäre unserer Erde* aus dem CCCI-422 gänzlich zu entfernen.

Ziel dieses Artikels war es, Argumente für die zuverlässige Interpretation der Personenfähigkeiten des CCCI-422 zu finden und Hinweise auf die Grenzen des Einsatzes des CCCI-422 zu geben. In der Itementwicklung sind wir von unterschiedlichen Inhaltsbereichen ausgegangen und haben deshalb auch eine fünfdimensionale Struktur des Testinstruments untersucht und für eine Gleichwertigkeit zum eindimensionalen Modell argumentiert. Als Limitation lässt sich hier jedoch anführen, dass zwar die Reihenfolge der Antwortalternativen randomisiert vorgegeben wurde, nicht jedoch die Abfolge der Items. Diese wurden nach Inhaltsbereich geclustert vorgegeben, also zuerst alle Items zu *Atmosphäre unserer Erde*, danach alle Items zu *Klima als System* und so weiter. Es kann also sein, dass die gute Passung des fünfdimensionalen Modells zum Teil auch auf die vorgegebene Reihenfolge der Items zurückzuführen ist und weniger auf die tatsächliche Zusammengehörigkeit der Items.

Prinzipiell sind aber auch andere Dimensionalisierungen des Testinstruments denkbar, etwa auf Basis unterschiedlicher Vorstellungen, Ideen oder der Unterscheidung in Vorstellungsfragen, Konzeptfragen und Faktenfragen. Auch diese Fragestellungen sollten in zukünftigen Studien untersucht werden. Zusammenhängend damit sollten auch Konsistenzüberprüfungen auf Basis der gewählten Alternativantworten durchgeführt werden, also inwiefern etwa die Vorstellung, dass Treibhausgase das Ozonloch vergrößern, konsistent über mehrere Items hinweg vertreten wird. Bisherige Forschung hat gezeigt, dass Lernende Vorstellungen oftmals stark vom Kontext oder Item abhängig vertreten (z. B. Chu und Treagust 2014).

Einen weiteren Diskussionspunkt wirft die Verteilung der mittleren Itemschwierigkeiten in den Abb. 6, 7 und 8 auf. Zentral ist hier, dass sich die Verteilung der Konzeptschwierigkeiten nur auf den aktuellen Wissensstand bezieht und damit keinen Anspruch darauf haben kann, typische Lernverläufe oder eine Learning Progression (Steedle und Shavelson 2009) darzustellen. Wir treffen diese Einschätzung insbesondere vor dem Hintergrund, dass die naturwissenschaftlichen Grundlagen des Klimawandels aktuell noch nicht so stark in Lehrplänen und Curricula verankert sind und daher auch davon auszugehen ist, dass in den Schulen eventuell noch keine oder wenig fachliche Instruktion zum Thema Klimawandel stattgefunden hat. Insbesondere zum Inhaltsbereich *Atmosphäre unserer Erde* lässt sich vermuten, dass sich die mittlere Itemschwierigkeit nach einer Instruktion zum Thema deutlich verschiebt. Diese Punkte könnten auch in zukünftigen Studien, etwa anhand von Instruktion zum Thema Klimawandel, genauer untersucht werden. Damit zusammenhängend gilt es auch die erhöhten Outfitwerte insbesondere in größeren Instruktionsstudien zu untersuchen und näher zu beleuchten. Obwohl wir die Nutzung von Rasch-Modellen zur Interpretation des CCCI-422 empfehlen, möchten wir dennoch eine weitere Limitation anführen, die sich im Rahmen der Interpretation der Testwerte anhand klassischer Testtheorie ergibt. So zeigen einige Items (tendenziell jene, die auch leicht erhöhte Outfitwerte aufweisen) eine niedrige Itemtrennschärfe (< 0,3), die in der Interpretation von klassischen Scores berücksichtigt werden sollte.

Als letzte Limitation der präsentierten Ergebnisse ist anzuführen, dass im Rahmen der Entwicklungsstudien kein paralleler Test zur Erhebung der Lesekompetenz der ProbandInnen eingesetzt wurde. Die Items des CCCI-422 selbst sind aus unserer Sicht tendenziell textlastig, was einerseits der Komplexität des Themas Klimawandels zugeschrieben werden kann, andererseits ist es für die Erhebung von Vorstellungen zumeist notwendig, diese auch zu beschreiben. In zukünftigen Studien sollte dieser Aspekt daher berücksichtigt werden oder es sollten diesbezüglich zusätzliche Validitätsargumente formuliert werden.

## Zusammenfassung und Ausblick

Klimawandel ist ein Thema, das vermehrt in den naturwissenschaftlichen Unterricht einziehen sollte. Obwohl Fachwissen nur eine kleine Rolle in Bezug auf nachhaltiges Handeln spielt, ist die Vermittlung eines grundlegenden Verständnisses der naturwissenschaftlichen Grundlagen des Klimawandels einerseits Aufgabe des naturwissenschaftlichen Unterrichts und andererseits hilft es dabei, widersprüchliche Darstellungen besser einordnen zu können (Tasquier und Pongiglione 2017). Wenn wir möchten, dass SchülerInnen über naturwissenschaftliche Grundlagen des Klimawandels lernen, sollte es uns auch möglich sein, Lernzuwächse zuverlässig zu messen. Deshalb haben wir den CCCI-422 entwickelt. In diesem Artikel haben wir die Entwicklung des CCCI-422 dargestellt und unterschiedliche Argumente für dessen Einsatz dargelegt. Durch das Vorliegen eines derartigen Testinstruments wird es nun möglich, auch im deutschsprachigen Raum zuverlässige Aussagen über das konzeptuelle Verständnis der naturwissenschaftlichen Grundlagen des Klimawandels von Lernenden ab der 8. Schulstufe zu treffen. Der CCCI-422 ermöglicht nun etwa, die in der Motivation aufgeworfenen Fragen nach der Ursache alternativer Lernendenvorstellungen und deren Entwicklung anhand von Interventionen zu untersuchen. Auch wenn Fachwissen nur eine Facette von *Climate Literacy* darstellt, so kann anhand des CCCI-422 nun auch untersucht werden, welche konkrete Rolle konzeptuelles Wissen (im Gegensatz etwa zu selbstberichtetem Wissen) über den Klimawandel für benachbarte Konstrukte wie Einstellungen zum Klimawandel, der Bewertungskompetenz (Ratzek und Höttecke 2021) oder dem kritischen Denken einnimmt. Wir möchten daher mit diesem Artikel auch dazu aufrufen, den CCCI-422 in zukünftigen Studien einzusetzen und zu nutzen. Wie bereits in der Diskussion angedeutet gibt es über die Messung von Lernzuwächsen hinaus noch viele weitere Fragestellungen, die sich mit diesem Concept Inventory in Zukunft beantworten lassen.

### Supplementary Information





## Anhang

**Table 5:**

Item	Konzept	Outfit	Infit
1	Atmosphäre unserer Erde	1,09	1,06
2	Atmosphäre unserer Erde	0,96	0,95
3	Atmosphäre unserer Erde	0,79	0,92
4	Atmosphäre unserer Erde	1,23	0,97
5	Atmosphäre unserer Erde	1,24	1,15
6	Atmosphäre unserer Erde	1,16	1,13
7	Klima als System	1,04	1,04
8	Klima als System	0,97	0,98
9	Klima als System	1,25	1,13
10	Klima als System	0,95	0,95
11	Klima als System	0,93	0,97
12	Klima als System	0,86	0,94
13	Klima als System	0,96	0,97
14	Klima als System	1,28	1,00
15	Kohlenstoffkreislauf	0,82	0,89
16	Kohlenstoffkreislauf	0,96	0,98
17	Kohlenstoffkreislauf	0,88	0,91
18	Kohlenstoffkreislauf	1,05	1,01
19	Kohlenstoffkreislauf	0,99	0,98
20	Kohlenstoffkreislauf	0,80	0,88
21	Unterschied Wetter – Klima	1,01	1,00
22	Unterschied Wetter – Klima	0,90	0,93
23	Unterschied Wetter – Klima	0,96	0,96
24	Unterschied Wetter – Klima	0,90	0,95
25	Unterschied Wetter – Klima	0,81	0,92
26	Unterschied Wetter – Klima	1,13	1,09
27	Unterschied Wetter – Klima	0,91	0,92
28	Treibhauseffekt	1,18	1,07
29	Treibhauseffekt	1,20	1,01
30	Treibhauseffekt	1,00	0,97
31	Treibhauseffekt	1,18	1,05
32	Treibhauseffekt	1,20	1,12
33	Treibhauseffekt	1,43	1,15
34	Treibhauseffekt	0,91	0,92
35	Treibhauseffekt	0,94	0,95
