# Supplementary material for: Development of the Concept Inventory CCCI-422 Regarding the Underlying Scientific Principles of Climate Change
Source: Z Didakt Nat Wiss. 2023 May 4;29(1):10. [Article in German] doi: 10.1007/s40573-023-00159-8 (PMC10159232; doi:10.1007/s40573-023-00159-8)
Supplement: Supplementary file 1 [file 40573_2023_159_MOESM1_ESM.pdf]

Climate Change Concept Inventory-422 (CCCI-422)

entwickelt

in einem Kooperationsprojekt

der Ruhr-Universität Bochum, der Universität Innsbruck und der Universität Graz.

Stand 15.03.2023

Ruhr-Universität Bochum	Universität Innsbruck	Universität Graz
**Rainer Wackermann**<br>**Carina Wöhlke**<br>**Hannes Lindemann**<br>**Kai Cardinal**<br>**Marko Jedamski**	**Thomas Schubatzky**	**Claudia Haagen-Schützenhöfer**

Der CCCI-422 besteht insgesamt aus 36 Items. Diese Items wurden vom Projektteam fünf unterschiedlichen Konzepten zugeordnet:

- **Die Atmosphäre unserer Erde**
- **Das Klima als System**
- **Der Kohlenstoffkreislauf**
- **Klima und Wetter**
- **Der Treibhauseffekt**

Alle Items sind Multiple-Choice Fragen im Single-Select-Format. Es ist sinnvoll, die Reihenfolge der Fragen so wie in diesem Dokument angeführt beizubehalten. Die Reihenfolge der Antworten kann beliebig variiert werden. Die Reihenfolge der Antworten in den Items ist in diesem Dokument so, dass die richtige Antwort immer Antwortalternative A ist.

CCCI-422 von Thomas Schubatzky, Rainer Wackermann, Claudia Haagen-Schützenhöfer, Carina Wöhlke, Hannes Lindemann, Kai Cardinal, Marko Jedamski ist lizenziert unter einer Creative Commons Namensnennung - Weitergabe unter gleichen Bedingungen 4.0 International Lizenz.

<table>
<tr><th colspan="3">Die Atmosphäre unserer Erde</th></tr>
<tr><td rowspan="5">A1</td><td colspan="2">Stell dir unsere Atmosphäre wie einen Behälter vor. Wo in diesem Behälter finden die für das Klima und Wetter relevanten Abläufe hauptsächlich statt?
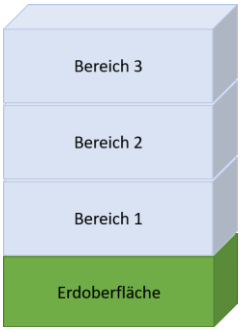
</td></tr>
<tr><td>A</td><td>Die für das Klima und Wetter relevanten Abläufe finden hauptsächlich im unteren Bereich 1, also dem erdbodennahen, statt.</td></tr>
<tr><td>B</td><td>Die für das Klima und Wetter relevanten Abläufe finden hauptsächlich im oberen Bereich 3, also dem weltraumnahen, statt.</td></tr>
<tr><td>C</td><td>Die für das Klima und Wetter relevanten Abläufe finden hauptsächlich im mittleren Bereich 2, also weder erdboden- noch weltraumnah, statt.</td></tr>
<tr><td>D</td><td>Die für das Klima und Wetter relevanten Abläufe finden in allen Bereichen gleich statt.</td></tr>
<tr><td colspan="3"></td></tr>
<tr><td rowspan="5">A2</td><td colspan="2">Die Erdatmosphäre besteht zu mehr als 90% aus zwei Bestandteilen. Welche zwei Gase sind das?</td></tr>
<tr><td>A</td><td>Stickstoff ($N2$) und Sauerstoff ($O2$)</td></tr>
<tr><td>B</td><td>Kohlenstoffdioxid ($CO2$) und Sauerstoff ($O2$)</td></tr>
<tr><td>C</td><td>Kohlenstoffdioxid ($CO2$) und Stickstoff ($N2$)</td></tr>
<tr><td>D</td><td>Kohlenstoffdioxid ($CO2$) und Wasserdampf ($H2O$)</td></tr>
<tr><td colspan="3"></td></tr>
<tr><td rowspan="6">A3</td><td colspan="2">Treibhausgase sind Bestandteile der Atmosphäre, die das Klima besonders beeinflussen. Wie hoch ist der Anteil dieser Treibhausgase in der Atmosphäre?</td></tr>
<tr><td>A</td><td>Weniger als 1%</td></tr>
<tr><td>B</td><td>Zwischen 1% und 5%</td></tr>
<tr><td>C</td><td>Zwischen 5% und 30%</td></tr>
<tr><td>D</td><td>Zwischen 30% und 55%</td></tr>
<tr><td>E</td><td>Über 55%</td></tr>
</table>

A4	**Welches Treibhausgas kommt am häufigsten in der Atmosphäre vor?**	
	A	**Wasserdampf (H_2O)**
	B	Kohlenstoffdioxid (CO_2)
	C	Methan (CH_4)
	D	Ozon (O_3)
A5	**Treibhausgase sind Bestandteile der Atmosphäre, die das Klima besonders beeinflussen. Wie hoch war der Anteil dieser Treibhausgase in der Atmosphäre VOR DER INDUSTRIALISIERUNG?**	
	A	**Weniger als 1%**
	B	Zwischen 1% und 5%
	C	Zwischen 5% und 30%
	D	Zwischen 30% und 55%
	E	Über 55%
A6	**Stell dir vor, der Anteil aller Treibhausgase bleibt ab heute konstant. Wie würde sich das Klima dann weiterentwickeln?**	
	A	**Das Klima würde sich in den nächsten 50 Jahren weiter erwärmen.**
	B	Das Klima würde sich nur in den nächsten Jahren weiter erwärmen.
	C	Das Klima würde sich in den nächsten 50 Jahren abkühlen.
	D	Das Klima würde in den nächsten 50 Jahren etwa so wie heute bleiben.
A7	**Wie lange bleibt Kohlenstoffdioxid (CO_2) durchschnittlich in der Atmosphäre?**	
	A	**Etwa 100 Jahre**
	B	Etwa 2 Monate
	C	Etwa 3 Jahre
	D	Etwa 5.000 Jahre
	E	Etwa 100.000 Jahre

Das Klima als System		
KS1	**Wenn das Klima wärmer wird, schmelzen Eis und Schnee, z. B. Gletscher. Wie wirken sich diese Schmelzvorgänge auf das Klima aus?**	
	A	**Das Klima wird schneller wärmer.**
	B	Das Klima wird langsamer wärmer.
	C	Das Klima wird insgesamt kälter.
	D	Das Klima wird dadurch nicht beeinflusst.
	E	Das ist nicht so einfach entscheidbar.
KS2	**Wenn das Klima wärmer wird, erwärmen sich die Ozeane. Die Ozeane nehmen dann weniger Kohlenstoffdioxid (CO_2) auf. Wie wirkt sich das auf das Klima aus?**	
	A	**Das Klima wird schneller wärmer.**
	B	Das Klima wird langsamer wärmer.
	C	Das Klima wird insgesamt kälter.
	D	Das Klima wird dadurch nicht beeinflusst.
	E	Das ist nicht so einfach entscheidbar.
KS3	**Wenn das Klima wärmer wird, gibt es mehr Wolken. Wie wirkt sich eine höhere Wolkendichte auf das Klima aus?**	
	A	**Das ist nicht so einfach entscheidbar.**
	B	Das Klima wird langsamer wärmer.
	C	Das Klima wird insgesamt kälter.
	D	Das Klima wird dadurch nicht beeinflusst.
	E	Das Klima wird schneller wärmer.

KS4	**Stell dir vor, die Eisschollen auf den Ozeanen werden weniger. Was wird dann passieren?**	
	A	**Weniger Sonnenlicht wird von der Erde in das Weltall reflektiert und die Temperatur auf der Erde steigt.**
	B	Mehr Sonnenlicht wird von der Erde in das Weltall reflektiert und die Temperatur auf der Erde sinkt.
	C	Mehr Sonnenlicht wird von der Erde in das Weltall reflektiert und die Temperatur auf der Erde steigt.
	D	Weniger Sonnenlicht wird von der Erde in das Weltall reflektiert und die Temperatur auf der Erde sinkt.
	E	Das Schmelzen von Eisschollen in den Ozeanen hat keinen Einfluss auf die Temperatur auf der Erde.
KS5	**Welche der genannten Teilbereiche der Erde beeinflussen das Klima und das Wetter auf der Erde?**	
	A	**die Atmosphäre, die Lebewesen und die Ozeane**
	B	die Atmosphäre und die Ozeane
	C	die Ozeane und die Lebewesen
	D	nur die Atmosphäre
	E	nur die Menschen
KS6	**Was trägt zur Verteilung von Wärmeenergie auf unserem Planeten bei?**	
	A	**Die Bewegung von Luft und die Strömung von Ozeanwasser tragen beide bei.**
	B	Nur die Bewegung von Luft trägt bei.
	C	Nur die Strömung von Ozeanwasser trägt bei.
	D	Die Bewegung von Luft und die Strömung von Ozeanwasser tragen beide nicht bei.
KS7	**Klimaveränderungen hat es schon immer gegeben. Was unterscheidet die Aktuelle von bisherigen Klimaveränderungen?**	
	A	**Die aktuelle Klimaerwärmung schreitet schneller voran als bisher.**
	B	Die aktuelle Klimaerwärmung ist größer als bisher.
	C	Die aktuelle Klimaerwärmung hat Auswirkungen auf die Menschheit.
	D	Die aktuelle Klimaerwärmung unterscheidet sich nicht von bisherigen Klimaveränderungen.

KS8	**Die verschiedenen Treibhausgase kommen in der Atmosphäre in unterschiedlicher Menge vor. Die Menge an Wasserdampf (H_2O) in der Atmosphäre ist weit größer als die von Kohlenstoffdioxid (CO_2). Warum ist Kohlenstoffdioxid (CO_2) dennoch hauptverantwortlich für den menschenverursachten Klimawandel?**	
	A	**Kohlenstoffdioxid (CO_2) beeinflusst den Anteil von Wasserdampf (H_2O) in der Atmosphäre.**
	B	Kohlenstoffdioxid (CO_2) sammelt sich in den oberen Schichten der Atmosphäre, Wasserdampf (H_2O) ist eher in Erdbodennähe.
	C	Wasserdampf (H_2O) kann von Pflanzen aufgenommen werden, Kohlendioxid (CO_2) aus fossilen Brennstoffen nicht.
	D	Kohlenstoffdioxid (CO_2) ist dichter als Wasserdampf (H_2O).
	E	Kohlenstoffdioxid (CO_2) zerstört die Ozonschicht in der Atmosphäre, Wasserdampf (H_2O) aber nicht.

**Der Kohlenstoffkreislauf**		
KK1	**Kohlenstoff (C) liegt in verschiedenen Formen vor, z.B. Kohle, Öl oder Kohlenstoffdioxid (CO_2). Wie hat sich die Gesamtmenge an Kohlenstoff (C) der Erde und in ihrer Atmosphäre seit den letzten 150 Jahren verändert?**	
	A	**Die Gesamtmenge an Kohlenstoff(C) ist gleichgeblieben.**
	B	Die Gesamtmenge an Kohlenstoff (C) ist etwas größer geworden.
	C	Die Gesamtmenge an Kohlenstoff (C) ist viel größer geworden.
	D	Die Gesamtmenge an Kohlenstoff (C) ist etwas kleiner geworden.
	E	Die Gesamtmenge an Kohlenstoff (C) ist viel kleiner geworden.
KK2	**Beim Verbrennen fossiler Brennstoffe gelangt der Kohlenstoff (C) dieser Brennstoffe als Kohlenstoffdioxid (CO_2) in unsere Atmosphäre. Kann dieser Kohlenstoff (C) aus dem Kohlenstoffdioxid (CO_2) irgendwann von einer Pflanze aufgenommen werden?**	
	A	**Ja, die Pflanzen können den Kohlenstoff (C) aus Kohlenstoffdioxid (CO_2) durch Photosynthese aufnehmen.**
	B	Ja, wenn es regnet, gelangt der Kohlenstoff (C) in Form von Kohlenstoffdioxid (CO_2) in den Boden und wird dort von Pflanzen aufgenommen.
	C	Nein, Kohlenstoffdioxid (CO_2) und die Pflanzen kommen nicht in Kontakt, weil das gasförmige Kohlenstoffdioxid (CO_2) nach oben steigt.
	D	Nein, der Kohlenstoff (C) aus der Verbrennung von fossilen Brennstoffen ist künstlich erzeugt und kann von Pflanzen nicht aufgenommen werden.
KK3	**Kommt Kohlenstoff (C) in dem Wasser der Ozeane vor?**	
	A	**Ja, weil Kohlenstoffdioxid (CO2) von den Ozeanen aufgenommen werden kann.**
	B	Nein, weil Ozeane nur aus Wasser bestehen.
	C	Ja, weil Wassermoleküle Kohlenstoff (C) enthalten.
	D	Nein, weil Flüssigkeiten keinen Kohlenstoffdioxid (CO2) enthalten können.

KK4	**Kohlenstoff (C) ist auf unserer Erde in den Ozeanen, den Lebewesen (Pflanzen und Tiere), den Eisflächen und der Atmosphäre gespeichert. In welchem dieser Teile ist am meisten Kohlenstoff (C) enthalten?**	
	A	**In den Ozeanen (Meere)**
	B	In den Lebewesen (Pflanzen und Tiere)
	C	In den Eisflächen (Grönland, Arktis und Antarktis)
	D	In der Atmosphäre (Luft)
KK5	**Vorgänge wie Photosynthese und Zellatmung ermöglichen einen Austausch von Kohlenstoff (C) zwischen Atmosphäre und Pflanzen. Welcher Vorgang beschreibt diesen Austausch vor dem menschenverursachten Klimawandel am besten?**	
	A	**Der Austausch von Kohlenstoff (C) zwischen Atmosphäre und Pflanzen war ungefähr ausgeglichen.**
	B	Der Kohlenstoff (C) sammelte sich in der Atmosphäre an.
	C	Der Kohlenstoff (C) sammelte sich in den Pflanzen an.
	D	Den Austausch von Kohlenstoff (C) gibt es erst seit dem aktuellen Klimawandel.
KK6	**Stell dir vor, ab heute nehmen die Ozeane kein Kohlenstoffdioxid (CO_2) aus der Atmosphäre mehr auf. Was passiert?**	
	A	**Der CO2-Gehalt in der Atmosphäre steigt stärker als bisher.**
	B	Der CO2-Gehalt in der Atmosphäre steigt weiterhin wie bisher.
	C	Der CO2-Gehalt in der Atmosphäre bleibt gleich.
	D	Der CO2-Gehalt in der Atmosphäre sinkt im Gegensatz zu jetzt.

**Klima und Wetter**		
KW1	**MeteorologInnen machen Aussagen über zukünftiges Wetter. Für welchen maximalen Zeitraum gibt es zuverlässige Wettervorhersagen?**	
	A	**Für einige Tage bis zu einer Woche.**
	B	Für bis zu drei Wochen.
	C	Für bis zu drei Monate.
	D	Für bis zu mehreren Jahren.
KW2	**Was ist Klima?**	
	A	**Klima fasst Wetter über einen längeren Zeitraum zusammen.**
	B	Klima ist das Wetter bzw. die Witterung an einem bestimmten Ort.
	C	Klima ist nur eine andere Bezeichnung für Wetter.
	D	Klima ist das, was wir spüren, wenn wir nach draußen gehen.
KW3	**Für welchen Zeitraum treffen MeteorologInnen üblicherweise Aussagen über das Klima?**	
	A	**Für ca. 30 Jahre**
	B	Für ca. 1 Woche
	C	Für ca. 3 Monate
	D	Für ca. 3 Jahre
KW4	**In welchem Zusammenhang stehen Wetter und Klima zueinander?**	
	A	**Klima fasst das Wetter über einen längeren Zeitraum zusammen. Klima ist somit eine Mittelung des Wetters.**
	B	Wetter und Klima beschreiben zwei unterschiedliche Modelle. Es gibt keinen Zusammenhang.
	C	Wetter und Klima beschreiben Wetterphänomene an einem Ort. Die beiden Begriffe beschreiben dasselbe.
	D	Das Wetter ist die Ursache für das Klima. Das Klima aber nicht die Ursache für das Wetter.

KW5	**Der Klimawandel hat unterschiedliche Auswirkungen auf unsere Erde. Welche Auswirkungen sind das?**	
	A	**Extreme Wetterereignisse treten häufiger auf. Die durchschnittliche Temperatur steigt.**
	B	Extreme Wetterereignisse treten häufiger auf. Die durchschnittliche Temperatur bleibt konstant.
	C	Extreme Wetterereignisse treten nicht häufiger auf. Die durchschnittliche Temperatur steigt.
	D	Extreme Wetterereignisse treten gleich häufig auf. Die durchschnittliche Temperatur bleibt konstant.
KW6	**Das Klima der Erde befindet sich in einem regelmäßigen Wechsel zwischen Warmzeiten und Eiszeiten. Was ist der mittlere Temperaturunterschied zwischen der letzten Eiszeit und heute?**	
	A	**4°C bis 5°C**
	B	1,5°C bis 2°C
	C	20°C bis 30°C
	D	40°C bis 50°C
KW7	**Wenn es im Sommer über einen längeren Zeitraum kaum regnet und dadurch weniger Wasser vorhanden ist als benötigt, spricht man von einem Dürresommer. Wie hängen die drei Dürresommer in Deutschland (2018, 2019, 2020) mit dem Klimawandel zusammen?**	
	A	**Dürren würden auch ohne den Klimawandel auftreten. Durch den Klimawandel treten Dürren häufiger und extremer auf.**
	B	Dürren sind eindeutig eine Folge des Klimawandels. Dürren werden durch den Klimawandel verursacht.
	C	Dürren sind eindeutig keine Folge des Klimawandels. Dürren würden auch ohne den Klimawandel auftreten.
	D	Dürren würden auch ohne den Klimawandel auftreten. Durch den Klimawandel werden Dürren seltener und schwächer.

**Der Treibhauseffekt**		
TE1	**Welche Aussage beschreibt den Treibhauseffekt auf der Erde am besten?**	
	A	**Sonnenstrahlung gelangt durch die Atmosphäre und erwärmt den Erdboden. Von der Erde abgegebene Wärmestrahlung wird von den Treibhausgasen in unserer Atmosphäre aufgenommen. Danach wird diese Wärmestrahlung unter anderem wieder in Richtung Erde abgestrahlt. Dadurch erwärmt sich die Erde stärker.**
	B	Sonnenstrahlung gelangt durch die Atmosphäre und erwärmt den Erdboden. Der Erdboden reflektiert diese Sonnenstrahlung. Diese Strahlung wird von den Treibhausgasen in unserer Atmosphäre wieder zur Erde reflektiert. Dadurch erwärmt sich die Erde stärker.
	C	Treibhausgase beschädigen die Ozonschicht in unserer Atmosphäre. Dadurch erzeugen und vergrößern sie das Ozonloch. Durch das Ozonloch kann mehr Sonnenstrahlung den Erdboden erreichen. Dadurch erwärmt sich die Erde stärker.
	D	Treibhausgase in unserer Atmosphäre bündeln die eintreffenden Sonnenstrahlen. Durch die gebündelten Sonnenstrahlen erwärmt sich die Erde stärker.
	E	Treibhausgase isolieren durch ihre hohe Dichte gut. Treibhausgase steigen zum Rand der Atmosphäre auf und verhindern so einen Wärmeaustausch der Erde mit dem Weltall. Dadurch erwärmt sich die Erde stärker.
TE2	**Von der Sonne wird Energie in unterschiedlichen Formen von Strahlung zur Erde transportiert. In welcher Form gelangt die meiste Energie auf die Erde?**	
	A	**In Form von sichtbarem Licht.**
	B	In Form von Wärmestrahlung.
	C	In Form von UV-Strahlung.
	D	In Form von radioaktiver Strahlung.
TE3	**Wie reagieren Treibhausgase in unserer Atmosphäre mit eintreffender sichtbarer Sonnenstrahlung?**	
	A	**Sie reagieren nicht mit sichtbarer Sonnenstrahlung.**
	B	Sie reflektieren die sichtbare Sonnenstrahlung.
	C	Sie nehmen die sichtbare Sonnenstrahlung auf.
	D	Sie binden die sichtbare Sonnenstrahlung.
	E	Sie bündeln sichtbare Sonnenstrahlung.

TE4	**Ein Teil der sichtbaren Sonnenstrahlung wird von der Erdoberfläche Richtung All reflektiert. Wie reagieren Treibhausgase in unserer Atmosphäre mit dieser reflektierten sichtbaren Sonnenstrahlung?**	
	A	**Sie reagieren nicht mit der sichtbaren Sonnenstrahlung.**
	B	Sie reflektieren die sichtbare Sonnenstrahlung.
	C	Sie nehmen die sichtbare Sonnenstrahlung auf.
	D	Sie binden die sichtbare Sonnenstrahlung.
TE5	**Die Erde gibt Wärmestrahlung ab. Wie reagieren Treibhausgase in unserer Atmosphäre mit dieser Wärmestrahlung?**	
	A	**Sie nehmen die von der Erde abgegebene Wärmestrahlung auf und geben sie wieder ab.**
	B	Sie reflektieren die von der Erde abgegebene Wärmestrahlung.
	C	Sie binden die von der Erde abgegebene Wärmestrahlung.
	D	Sie reagieren nicht mit der von der Erde abgegebenen Wärmestrahlung.
	E	Sie streuen die von der Erde abgegebene Wärmestrahlung.
TE6	**Wie stark ist die mittlere Temperaturerhöhung der Erde durch den natürlichen Treibhauseffekt im Vergleich zum menschenverursachten Treibhauseffekt?**	
	A	**Die mittlere Temperaturerhöhung durch den natürlichen Treibhauseffekt ist höher als durch den menschenverursachten Treibhauseffekt.**
	B	Es gibt keinen natürlichen Treibhauseffekt.
	C	Die mittlere Temperaturerhöhung durch den natürlichen Treibhauseffekt ist geringer als durch den menschenverursachten Treibhauseffekt.
	D	Die mittlere Temperaturerhöhung durch den natürlichen Treibhauseffekt ist in etwa gleich hoch wie durch den menschenverursachten Treibhauseffekt.
TE7	**Unterschiedliche Oberflächen können unterschiedlich viel sichtbare Sonnenstrahlung reflektieren. Welche Oberfläche reflektiert am meisten Sonnenstrahlung?**	
	A	**Schnee**
	B	Ozeane
	C	Ackerböden
	D	Grünflächen

TE8	**Wie groß ist die Erhöhung der mittleren Temperatur der Erde durch den menschenverursachten Treibhauseffekt aktuell?**	
	A	**1°C – 2°C**
	B	0°C
	C	2°C – 10°C
	D	Über 10°C
